# Impaired cAMP processivity by phosphodiesterase-protein kinase A complexes in acrodysostosis

**DOI:** 10.3389/fmolb.2023.1202268

**Published:** 2023-09-21

**Authors:** Varun Venkatakrishnan, Abhijeet Ghode, Nikhil K. Tulsian, Ganesh S. Anand

**Affiliations:** ^1^ Department of Chemistry, Pennsylvania State University, University Park, PA, United States; ^2^ Department of Biological Sciences, National University of Singapore, Singapore, Singapore; ^3^ The Huck Institutes of the life sciences, Pennsylvania State University, University Park, PA, United States

**Keywords:** acrodysostosis, protein kinase A, RIα, phosphodiesterase, signal termination, allostery

## Abstract

Acrodysostosis represents a group of rare genetic disorders characterized by defective skeletal development and is often accompanied by intellectual disabilities. Mutations in the 3′5′cyclic AMP (cAMP)-dependent protein kinase (PKA) type I regulatory subunit isoform α (RIα) and phosphodiesterase (PDE) PDE4D have both been implicated in impaired PKA regulation in acrodysostosis. How mutations on PDEs and RIα interfere with the regulation of cAMP-PKA signaling is not understood. cAMP-PKA signaling can be described in two phases. In the activation phase, cAMP binding to RIα dissociates the free C-subunit (Catalytic subunit). PDEs hydrolyze cAMP bound to RIα, priming the cAMP-free RIα for reassociation with the C-subunit, thereby completing one PKA activation cycle. Signal termination is thus critical for resetting PKA to its basal state and promoting adaptation to hormonal hyperstimulation. This proceeds through formation of a transient signal termination RIα: PDE complex that facilitates cAMP channeling from the cAMP-binding domain of RIα to the catalytic site of PDE. Signal termination of cAMP-PKA proceeds in three steps: Step 1) Channeling: translocation of cAMP from the CNB of RIα to the PDE catalytic site for hydrolysis. Step 2) Processivity: binding of free cAMP from the cytosol at both CNBs of RIα. Step 3) Product (5′AMP) release from the PDE hydrolysis site through competitive displacement by a new molecule of cAMP that triggers subsequent activation cycles of PKA. We have identified the molecular basis for two acrodysostosis mutants, PDE (PDE8 T690P) and RIα (T207A), that both allosterically impair cAMP-PKA signal termination. A combination of amide hydrogen/deuterium exchange mass spectrometry (HDXMS) and fluorescence polarization (FP) reveals that PDE8 T690P and RIα T207A both blocked processive hydrolysis of cAMP by interfering with competitive displacement of product 5′AMP release from the nucleotide channel at the end of each round of cAMP hydrolysis. While T690P blocked product 5′AMP release from the PDE, T207A greatly slowed the release of the substrate from RIα. These results highlight the role of processivity in cAMP hydrolysis by RIα: PDE termination complexes for adaptation to cAMP from GPCR hyperstimulation. Impairment of the signal termination process provides an alternate molecular basis for acrodysostosis.

## 1 Introduction

**FIGURE 1:**
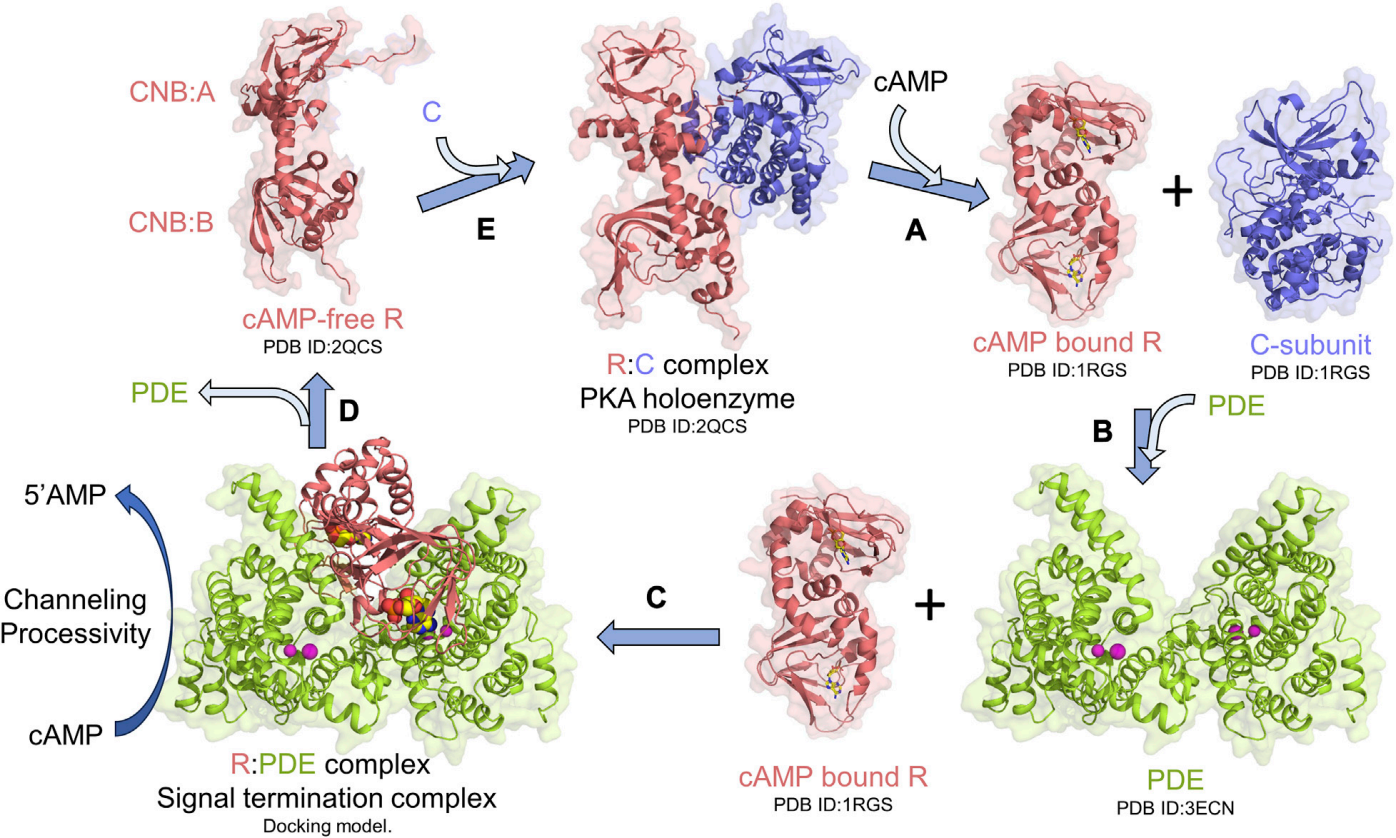
PKA signalosome. Simplified monomeric model for the PKA signalosome cycle. **(A)** cAMP binding to the R-subunit in the PKA holoenzyme complex activates PKA by releasing the C-subunit. **(B)** To terminate PKA, PDEs interact with cAMP-bound R through formation of (C) signal termination complex [Molecular docking model from Krishnamurthy et al. (2014)] that hydrolyzes cAMP bound to R through channeling of cAMP from CNB **(A and B)** of R to the PDE hydrolysis site. This complex also facilitates processive hydrolysis of cAMP from bulk solution to generate 5′AMP. **(D)** Signal termination complex dissociates resulting in cAMP-free R which then **(E)** reassociates with C to reform the PKA holoenzyme and reset PKA signaling.

Protein kinase A (PKA) is an essential and ubiquitous signaling enzyme that relays hormonal signals conveyed through membrane-anchored G-protein-coupled receptors (GPCRs) to intracellular effectors (Sassone-Corsi, 2012). PKA activation upon hormonal stimulation profoundly alters gene expression and cellular metabolism (Shabb, 2001). Within cells, PKA is organized into localized signaling modules referred to as signalosomes, which promote the spatiotemporal control of PKA signaling (Baillie et al., 2005; Surdo et al., 2017; Kar et al., 2021). PKA activation is regulated by the second messenger 3′5′cyclic adenosine monophosphate (cAMP), which is generated upon hormonal stimulation of GPCRs, that subsequently activates adenylyl cyclases to catalyze the synthesis of cAMP from ATP (Sassone-Corsi, 2012). Activation of PKA occurs when cAMP binds to tandem cyclic nucleotide-binding domains (CNB:A and B) of the regulatory subunit (R) of PKA, thereby unleashing the PKA catalytic subunit (C) to phosphorylate its numerous downstream targets (Herberg et al., 1994; Shabb, 2001; Taylor et al., 2005; Das et al., 2007; Kim et al., 2007) (Figure 1A). To reset PKA signaling, phosphodiesterases (PDEs) bind and hydrolyze cAMP bound to R through the formation of an R:PDE signal termination complex (Moorthy et al., 2011a; Krishnamurthy et al., 2013) (Figure 1B). The R:PDE complex shows higher PDE catalysis than PDE alone and facilitates cAMP hydrolysis through translocation of cAMP from the high-affinity cAMP-binding pocket within the CNBs of R directly to the PDE hydrolysis site through a channel that is formed across the R:PDE interface (Krishnamurthy et al., 2014; Tulsian et al., 2017) (Figure 1C). R:PDE facilitates processive hydrolysis of bursts of cAMP generated as a result of GPCR hyperstimulation to produce 5′adenosine monophosphate (5′AMP), thereby leading to rapid PKA signalosome reset to the inactive holoenzyme state (Tulsian et al., 2017; Tulsian et al., 2020) (Figures 1C–E). The R-PDE complex thus forms a basis for signal adaptation in cAMP-PKA signaling (Tulsian et al., 2020).

There are two non-redundant R mammalian isoforms— RI and RII with subtypes α and β (RIα, RIβ, RIIα, and RIIβ) (Skalhegg and Tasken, 2000; Vigil et al., 2004; Taylor et al., 2005). RIα is the most essential isoform of the PKA R-subunit with high binding affinities for cAMP (K_d_∼2–60 nM) and the C subunit (K_d_ = 0.2 nM) (Herberg et al., 1994; Amieux and McKnight, 2002; Kim et al., 2007). RIα mediates transient interactions with multiple PDEs and has been demonstrated to bind PDE8 to terminate cAMP-PKA signaling (Krishnamurthy et al., 2014; Tulsian et al., 2017). Termination proceeds through nucleotide channeling in the RIα:PDE8 complex (Krishnamurthy et al., 2014; Tulsian et al., 2017; Tulsian et al., 2020).

Aberrant cAMP-PKA signaling is implicated in disorders such as acrodysostosis, Carney complex, and Cushing’s syndrome (Kirschner et al., 2000; Silve et al., 2012a; Walker et al., 2019). Acrodysostosis (ACRDYS) represents a group of rare genetic disorders characterized by heterogeneous clinical features ranging from skeletal abnormalities such as brachydactyly, facial dysostosis, and nasal hypoplasia to intellectual and behavioral disabilities (Silve et al., 2012a; Silve et al., 2012b). Genetic analysis and exome sequencing identified missense mutations on *prkara1* and *pde4d* genes in patients diagnosed with ACRDYS (Linglart et al., 2012; Elli et al., 2016). ACRDYS-causing mutations on RIα have been identified to localize predominantly to the CNBs, some of which severely reduce the affinity of cAMP for RIα. Mutations on RIα are associated with hormone resistance and thereby classified as Type I ACRDYS (ACRDYS1) causing mutations (Linglart et al., 2011). Hormone resistance arises when cAMP-generated from upstream hormonal stimulation is insufficient to activate PKA at concentrations within the range of the binding constants of cAMP for PKA generated *in vitro* (Herberg et al., 1994).

**FIGURE 2:**
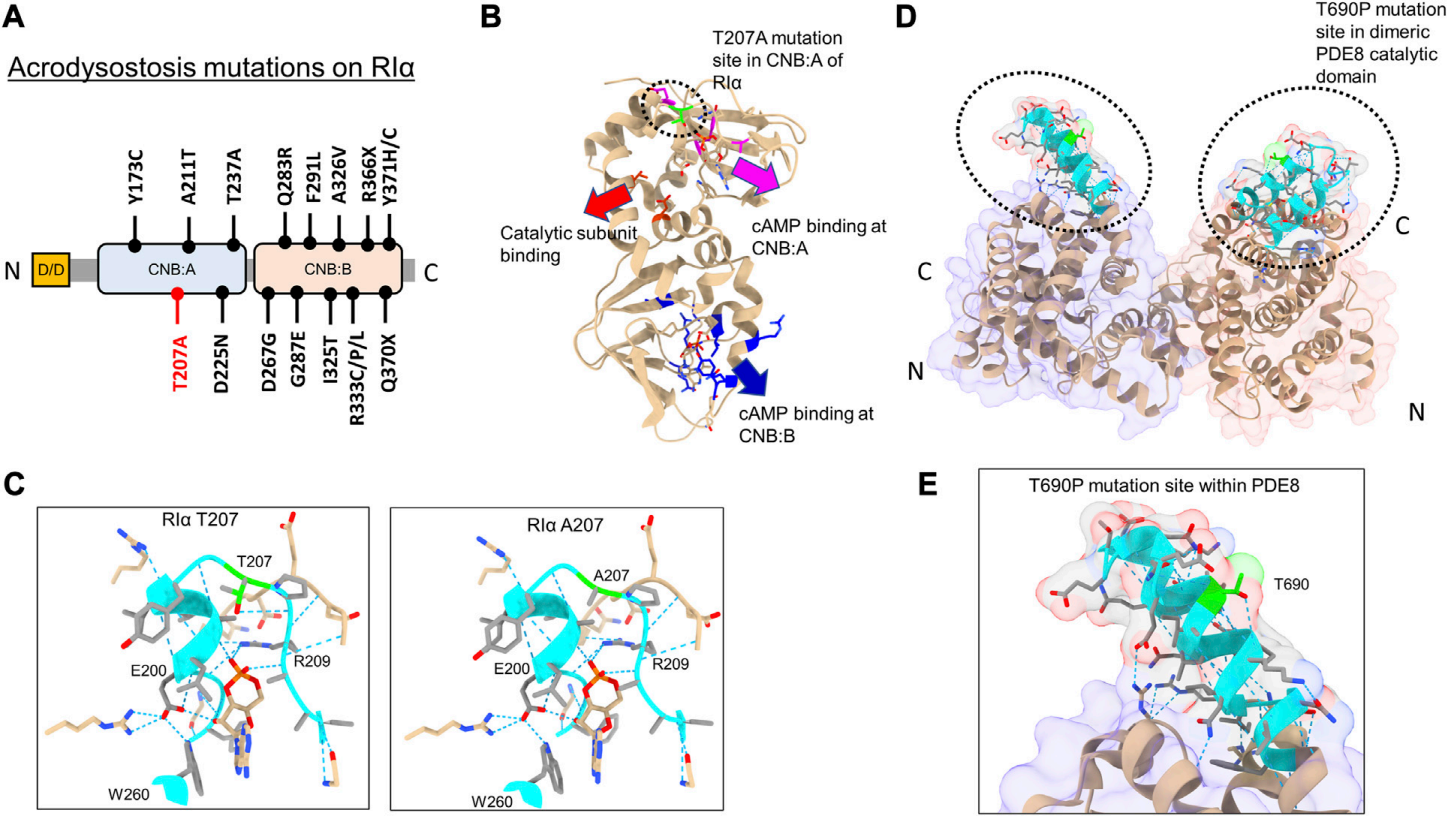
Acrodysostosis (ACRDYS) mutations on RIα and PDE8. **(A)** Domain organization of RIα with ACRDYS mutations highlighted. D/D-docking and dimerization domain, CNB—cyclic nucleotide binding domain. T207A is highlighted in red. **(B)** ACRDYS mutations highlighted on the structure of RIα (PDB ID:1RGS). Mutations impacting cAMP binding at CNB:A (magenta), CNB:B (blue), and PKA catalytic subunit binding (red) are highlighted. The T207A mutation site is highlighted in green. **(C)** Closeup of the cAMP binding site (phosphate binding cassette) of CNB:A in RIα highlighting the T207A mutation site and other residues critical for binding cAMP. The rotamer function in ChimeraX was used to model RIα T207A mutation and its impacts on H-bonding in cAMP-bound RIα (T207A (right) in comparison to WT RIα (left). **(D)** Structure of PDE catalytic domain dimer showing theT690 locus (PDB ID:3ECN). **(E)** Closeup of the T690P mutation within the RIα binding interface on PDE8(cyan). Dashed blue lines between residues denote H-bonds. Regions around the mutation site are highlighted in cyan, and the mutation site is in lime.

Of the 15 mutations identified in RIα, only five sites are localized within the CNB:A site (Elli et al., 2016) (Figures 2A, B). Although CNB:A contributes a bulk of the interaction interface with C in the PKA-I holoenzyme, only two ACRDYS1 mutation sites—T237 in the C-helix and D267 in the N3A helix of CNB:B—span the R:C interface, while other mutations are distal to the cAMP-binding sites (Bruystens et al., 2016; Chandramohan et al., 2017) (Figures 2A, B). This indicates that not all mutations associated with ACRDYS can be attributed entirely to impairment of cAMP binding or disruption of R:C interactions. We hypothesize that some mutations form the basis of acrodysostosis by disrupting signal termination*.* For instance, ACRDYS-causing mutations on the cAMP-specific PDE4D enhance the rates of cAMP hydrolysis (Linglart et al., 2012; Briet et al., 2017). This decreases the localized cAMP concentration and consequently reduces the sensitivity of PKA for cAMP. This process occurs independently of hormone activation and is accordingly classified as type II ACRDYS or ACRDYS without hormone resistance (ACRDYS2) (Silve et al., 2012b; Linglart et al., 2012).

In this study, we assessed the impact of PDE8 T690P (ACRDYS2) and RIα T207A (ACRDYS1) upon signal termination in PKA. Both mutations were first identified and described in 2016 during genetic screening of patients with ACRDYS (Elli et al., 2016). Although T207 is present within the phosphate-binding cassette (PBC) of CNB:A in RIα and is highly conserved across CNB:A from RIα, it does not mediate orthosteric contacts with cAMP (Su et al., 1995) (Figures 2B, C). T207 is a part of the RIα:PDE8 interface determined by HDXMS (Krishnamurthy et al., 2014), suggesting that this mutation might disrupt the RIα:PDE8 complex. We also examined the effects of an ACRDYS mutation in PDE4D (T594P) by characterizing an equivalent mutant PDE8 T690P on RIα:PDE8 interactions and cAMP processivity. Interestingly, T690 is distal to the catalytic site of PDE8 (Figures 2D, E; Supplementary Figure S1), is conserved across the PDE superfamily and is part of the RIα:PDE8 binding interface (Wang et al., 2008; Krishnamurthy et al., 2014). PDE4D and PDE8 are both cAMP-specific PDEs that share high sequence and structural homology (Supplementary Figure S1). RIα has been shown to interact with all PDEs, including PDE4, with the dynamics and kinetics of signal termination comparable to RIα:PDE8 complexes (Krishnamurthy et al., 2014; Tulsian et al., 2017; Tulsian et al., 2020).

cAMP signal termination consists of the following three steps: Step 1) Channeling: translocation of cAMP from the CNB of RIα to the PDE catalytic site for hydrolysis. Step 2) Processivity: binding of free cAMP from the cytosol at both CNBs of RIα. Step 3) Product (5′AMP) release from the PDE hydrolysis site through competitive displacement by new cAMP for a subsequent activation cycle. Here, we describe the impact of two ACRDYS mutations, PDE8 T690P and RIα T207A, on signal termination by applying two complementary solution approaches—amide hydrogen deuterium exchange mass spectrometry (HDXMS) and fluorescence polarization—to track changes in the proteins and ligands through signal termination. Our results reveal that both PDE8 T690P and RIα T207A disrupted the process of signal termination at two separate steps. Product 5′AMP release was greatly reduced in PDE8 T690P, and channeling was disrupted in RIα T207A. Both mutations thus impaired PKA signalosome responses to hyperstimulation of GPCRs and identified a novel molecular basis for acrodysostosis.

## 2 Methods

### 2.1 Site-directed mutagenesis of RIα and the PDE8 catalytic domain

Site-directed mutagenesis was carried out on full-length RIα to generate the T207A (A→G) point mutant using a forward primer: 5′-TGA TTT ACG GGG CCC CTC GA-3′ and reverse primer: 3′-TCG AGG GGC CCC GTA AAT CA-5’ (Integrated DNA technologies, Singapore). The PDE8A catalytic domain (472–829) construct was used to generate the T690P (A→C) point mutant using a forward primer: 5′-CTG GCG CCG CTG GAG GAA AAT GG-3′ and a reverse primer: 3′-TCC AGC GGC GCC AGC GGT TTA TT-5’. Point mutants were prepared with oligonucleotide primers for RIα T207A and PDE8c T690P using the Q5 high-fidelity DNA polymerase kit (New England Biolabs, Canada) as per the manufacturer’s instructions, and PCR products were validated by sequencing (AIT Biotech, Singapore).

### 2.2 Recombinant protein expression and purification

Full-length wild-type (WT) RIα and RIα T207A constructs were cloned into the pRSETA vector and overexpressed in *E. coli* BL21 DE3 (Thermo Scientific, United States) (Tulsian et al., 2017). Cells were induced at OD_600_ ∼0.6 with 0.5 mM IPTG and grown overnight at 18°C under constant shaking prior to harvesting the cell pellets. Recombinant RIα was purified by His-tag affinity chromatography using TALON resin (Takara Bio, United States) and eluted in a buffer containing 20 mM Tris-Cl pH 7.5, 150 mM NaCl, 250 mM imidazole, and 1 mM β-ME. The protein was enriched by size-exclusion chromatography in a HiLoad 16/60 Superdex 200 prep grade column (AKTA FPLC, Cytiva, United States/Enrich 650SEC column, BioRad, United States) and stored in buffer containing 20 mM Tris-Cl pH 7.5, 150 mM NaCl, and 1 mM β-ME.

The catalytic domain of wild-type PDE8A1 (henceforth referred to as PDE8 WT) and PDE8 T690P constructs were cloned into pET-Duet1 and overexpressed in *E. coli* BL21 DE3 (Thermo Scientific, United States) by induction at OD_600_ ∼0.6 with 0.5 mM IPTG. Recombinant PDE8 was purified via an unfolding–refolding process (Yan et al., 2009; Tulsian et al., 2017). PDE8 was extracted from the inclusion body pellet containing recombinant protein by denaturation in 0.1 M Tris-Cl pH 8.0 containing 6 M GdnHCl for 12 h at 25°C and purified by His-tag affinity chromatography using TALON (Takara Bio, United States) resin and eluted with a buffer containing 0.1 M Tris pH 8.0, 0.5 M imidazole, 6 M urea, and 0.5 M L-Arginine. The denatured protein was then refolded in a buffer containing 0.5 M Tris pH 7.0, 30% glycerol, 0.7 M L-Arginine, 10 mM NaCl, 20 mM MgCl_2_, 20 mM MnCl_2_, 1 mM KCl, 20 μM ZnSO_4_, and 10 mM DTT for 4 days. Refolded PDE8 was enriched by hydroxyapatite chromatography (BioGel HTP hydroxyapatite, BioRad, United States) and eluted in a buffer containing 50 mM Tris-Cl pH 7.0, 500 mM KH_2_PO_4_, and 5 mM β-ME. The phosphate was dialyzed out against 20 mM Tris-Cl pH 7.5, 50 mM NaCl, and 5 mM β-ME. Refolded PDE8 from the dialysis fractions was purified by anion-exchange chromatography using a MonoQ 5/50 GL Column (Cytiva, United States) eluting in a gradient of NaCl (50 mM–2 M) in 20 mM Tris-Cl pH 7.5, 5 mM MgCl_2_, and 5 mM β-ME followed by size-exclusion chromatography (HiLoad 16/60 Superdex 200 prep grade column, Cytiva, United States/Enrich 650SEC column, BioRad, United States) and stored in a buffer containing 20 mM Tris-Cl pH 7.5, 50 mM NaCl, 5 mM MgCl_2_, and 5 mM β-ME. The expression and purity of PDE8 (WT and T690P) and RIα (WT and T207A) were assessed by sodium dodecyl sulfate-polyacrylamide gel electrophoresis and quantified by Bradford assay and size-exclusion chromatography (Supplementary Figure S2).

### 2.3 Phosphodiesterase assay

Catalytic activity of refolded wild-type (WT) PDE8 and PDE8 T690P was evaluated using a BIOMOL green colorimetric phosphodiesterase assay (Enzo Life Sciences, NY, United States). This is a linked assay that indirectly measures the product 5′AMP generated by hydrolysis of cAMP using a modified malachite green reagent that reacts with free phosphate generated by the action of 5′Nucleotidase on the PDE hydrolysis product—5′AMP. We incubated (1 nM) PDE8 WT and PDE8 T690P with varying concentrations of cAMP ranging from 0.25–10 μM to encompass the previously reported K_M_ values (Yan et al., 2009; Tulsian et al., 2017). Reactions were carried out in triplicate in 96-well flat-bottom microtiter plates in 50 μL reaction volumes for 15 min at 25°C. The assays were performed in a buffer containing 20 mM Tris-Cl pH 7.5, 50 mM NaCl, 5 mM MgCl_2_, 20 μM ZnSO_4_, and 5 mM β-ME. The reactions were quenched by the addition of 100 μL Biomol green reagent, and the wells were analyzed in a SpectraMax plate reader (Molecular Devices, CA, United States) at 620 nm, and the calculations were fit to Michaelis–Menten kinetics, and K_M_, V_max_, and k_cat_ values were determined by GraphPad (San Diego, United States).

### 2.4 Amide hydrogen–deuterium exchange mass spectrometry (HDXMS)

HDXMS experiments were carried out to map the conformational effects of RIα T207A and PDE8 T690P on nucleotide binding and signal termination of PKA. All deuterium exchange reactions were carried out by adding 3 μL of the sample to 27 μL deuterium labeling buffer (99% D_2_O, 20 mM Tris-Cl pH 7.5, 50 mM NaCl, and 5 mM β-ME) for the final sample concentration of 1 μM and final deuteration of 90%. All deuterium exchange buffers for PDE8 were supplemented with 5 mM MgCl_2_ and 20 μM ZnSO_4_. To measure the effect of RIα T207A and PDE8 T690 on nucleotide channeling in RIα:PDE8, deuterium labeling buffer was supplemented with 330 μM cAMP, 3 mM 5′AMP, or as indicated. HDXMS of mutant signal termination complexes was carried out by complexation of PDE8 with RIα at a ratio of 2:1. RIα T207A was complexed with WT PDE8, and RIα was complexed with PDE8 T690P to characterize the ACRDYS signal termination complexes. Although an exact affinity for PDE8 and RIα has not been reported, our previous results of the mapping interactions of RIα with a PDE8 homolog, RegA, have estimated a K_D_ ∼0.1 μM (Moorthy et al., 2011b). Under our experimental conditions and protein concentrations for both HDXMS and FP, there would be complete complexation of PDE8 with RIα. Deuterium exchange was carried out in triplicates at 25°C for 1-, 10-, and 30-min labeling times. The reaction was quenched by the addition of 20 μL chilled 0.1% trifluoroacetic acid to lower the pH to 2.5.

Quenched samples were injected into an ACQUITY nano-UPLC HDX sample manager (Waters, Milford, United States) and digested into peptides in an immobilized pepsin column (Poroszyme, ABI, Foster city, United States) under a continuous flow rate of 100 μL/min in 0.1% formic Acid. Peptides were then trapped in a VanGuard trap column (Waters, Milford, United States) and loaded onto an ACQUITY BEH C-18 reverse-phase chromatography column (Waters, Milford, United States) and eluted under a 8%–40% gradient of acetonitrile in 0.1% formic acid at 40 μL/min pumped from an ACQUITY Binary Solvent Manager (Waters, Milford, United States). Peptides were ionized by ESI and analyzed in a Synapt G2-Si mass spectrometer (Waters, Milford, United States) in the MS^E^ mode as previously described (Tulsian et al., 2017). The mass spectrometer was calibrated continuously with the calibrant 200 fmol/μL Glu-Fibrinopeptide B at a flow rate of 2 μL/min. The total run time of LC-MS was 13 min consisting of a 3-min pepsin digestion step followed by a 10-min separation and acquisition.

### 2.5 HDXMS data analysis

Pepsin-proteolyzed fragment peptides from undeuterated controls were identified using ProteinLynx Global Server 3.0 (PLGS) using amino acid sequences for RIα (P00514) and PDE8 (O60658) obtained from UniProtKB. A sequence of porcine pepsin was not included in the search since immobilized pepsin was used. Peptide searches were performed by selecting non-specific protease representing pepsin with the number of missed cleavage sites—1, false discovery rate—4, low energy counts—250.0, and elevated energy counts—100.0. The sequence for PLGS was changed from T207 to A207 for RIα T207A and T690 to P690 for PDE8 T690P mutants. Deuterium exchange was analyzed using DynamX v3.0 (Waters, Milford, MA, United States), and peptides were filtered with cutoffs for minimum intensity—5000, minimum peptide length—5, maximum peptide length—25, mass tolerance—10 ppm, minimum products—1, and file threshold—2. Deuterium exchange values (Da) were calculated by subtracting the centroids of deuterated mass envelopes from the centroids of corresponding undeuterated mass envelopes. Deuterium exchange profiles comparing two different states were plotted in difference plots and mapped onto a molecular docking model generated from HDXMS analyses of the WT RIα:PDE8 complex described by Krishnamurthy et al. (2014); Krishnamurthy et al. (2014). The mass spectrometry data has been deposited to the ProteomeXchange Consortium via the PRIDE partner repository. The accession code for the HDXMS data is PXD045088 HDXMS methods and analysis are detailed in Supplementary Figure S1.

Bimodal deconvolution for monitoring cAMP release from RIα T207A in RIα T207A:PDE8 complexes were performed using HX-Express v3.0 (Weis et al., 2006; Guttman et al., 2013). Two overlapping peptides were identified from CNB:A (202–221, 206–221) and CNB:B (329–336, 330–336), respectively, that each showed spectral broadening upon deuterium exchange at longer times (t_ex_ > 10 min) (Weis et al., 2006). Binomial fitting was applied to the mass spectra for these peptides. Mass spectra displaying differences between the theoretical and experimental centroids upon binomial fitting were then used for application of bimodal analysis. Based on this, bimodal deconvolution was applied to all the aforementioned four peptides showing large differences between theoretical and experimental centroid values. These were observed for the binomial fits at deuterium exchange time t > 10 min. Two parameters were chosen to distinguish deconvolved bimodal spectra from spectral broadening. 1) *p*-value which is determined from an f-test compares the fit of two binomial spectra *versus* a single binomial to the experimental spectra. A *p*-value <0.05 was considered statistically significant for bimodal assignment. Mass spectral envelopes showing *p* > 0.05 were not deconvolved. 2) A confidence interval >95% in the regression metric, which measures the confidence of fit of the bimodal spectra (double binomial) to the experimental spectral envelope, was considered statistically significant.

To account for overfitting of bimodal spectra attributable to differences in signal-to-noise ratios, two additional secondary parameters were evaluated. 1) The Delta Chi metric which measures the improvement in the spectral fit when using a double binomial instead of a single binomial and 2) the separation metric, which measures the differences in centroids of the deconvolved lower and higher exchanging envelopes, where a separation >1.5 Da was considered statistically significant, indicative of near-baseline resolved bimodal spectra. Secondary parameters were considered for spectra with 0.02 < *p* < 0.05 to ensure there was no overfitting of the data. The results of the bimodal analysis are summarized in Table 1. Bimodals were not identified in deuterium exchange of RIα: T207A: PDE in excess cAMP, indicating the absence of bimodal spectral envelopes. This established the robustness of the bimodal deconvolutions that were evident only for RIα T207A: PDE complexes at times t_ex_>10 min.

**TABLE 1:** cAMP release from CNB sites from RIα T207A:PDE8. Bimodal analysis of overlapping spectra from CNB:A and CNB:B quantifying release of endogenous cAMP in RIα T207A:PDE8 complexes. A—the high *p*-values for peptides 329–336 and overlapping peptides 330–336 classify it as spectral broadening. B—mass spectra for peptides 207–222 at t_ex_ = 10 showed a low signal-to-noise ratio (S/N < 250), and hence bimodal deconvolution was not possible.

cAMP binding site on RIα T207A	Peptide	Spectral class	Deuteration time tex (min)	cAMP-bound state (left)		CAMP-free state (right)	
				Uptake (Da)	Left (%)	Uptake (Da)	Right (%)
CNB:A PBC	202–221 ALIYGAPRAATVKAKTNVKL	Bimodal (*p* < 0.01)	10 min	2.8 ± 0.05 Da	85.6% ± 3%	7.79 ± 0.04 Da	14.4% ± 3%
			30 min	3.32 ± 0.11 Da	65% ± 1.7%	8.65 ± 0.03 Da	35% ± 1.7%
	206–221 GAPRAATVKAKTNVKL	Bimodal (*p* < 0.01)	10 min (B)	-NA-	-NA-	-NA-	-NA-
			30 min	1.97 ± 0.09 Da	68% ± 2.8%	6.45 ± 0.16 Da	32% ± 2.8%
CNB:B PBC	329–336 MNRPRAAT	Broadening (0.01 < *p*< 0.05) (A)	10 min	-NA-		-NA-	
			30 min				
	330–336 NRPRAAT	Broadening (*p* > 0.05)	10 min	-NA-		-NA-	
			30 min				

### 2.6 Monitoring RIα:PDE8 T690P complexes by fluorescence polarization spectroscopy (FP)

To monitor cAMP channeling in RIα:PDE8 T690P complexes, two fluorescently labeled analogs of cAMP—PDE8 hydrolyzable 2’-[fluo-AHC]-cAMP (2’-fl-cAMP) and non-PDE8 hydrolyzable 8-[fluo]-cAMP (8-fl-cAMP) (Biolog, Germany) (Schwede et al., 2017)—were used. Polarization values provide a readout of relative molecular weights as larger protein complexes generate higher FP readings than smaller proteins because fluorophores bound to proteins tumble at slower rates as opposed to fast tumbling in unbound fluorophores (Rossi and Taylor, 2011). Approximately 1 μM wild-type RIα was incubated with molar excess (20 μM) of each of the analogs for 24 h at 4°C under constant shaking. Excess unbound ligands were removed by size-exclusion chromatography (HiLoad 16/60 Superdex 200 prep grade column, Cytiva, United States). Approximately 2 μM PDE8 T690P was added to the cAMP analog-bound RIα to facilitate complexation. FP reactions were carried out in black, flat-bottom 96-well plates (Greiner Bio-One, United States), and readings were measure on a Synergy 4 microplate reader (BioTek, United States) at a calculated G-factor of 0.87. Both analogs have excitation and emission wavelengths of 484 nm and 524 nm, respectively. Excess cAMP (25 or 100 μM) was added at the 20-min time-point of the FP experiment to monitor competitive displacement of the fluorescent analogs. Control experiments were carried out using analog-bound RIα and RIα-PDE8 T690P complexes without the addition of excess cAMP. To monitor processive hydrolysis, 2 μM PDE8 T690P was added at the 20-min time-point to RIα saturated with excess cAMP (100 μM). Control experiments were carried out with wild-type PDE8. All reactions were carried out in triplicate. Data processing and analysis were performed in MS Excel 2013.

## 3 Results

### 3.1 Complexation with RIα is similar in WT and PDE8 T690P

**FIGURE 3:**
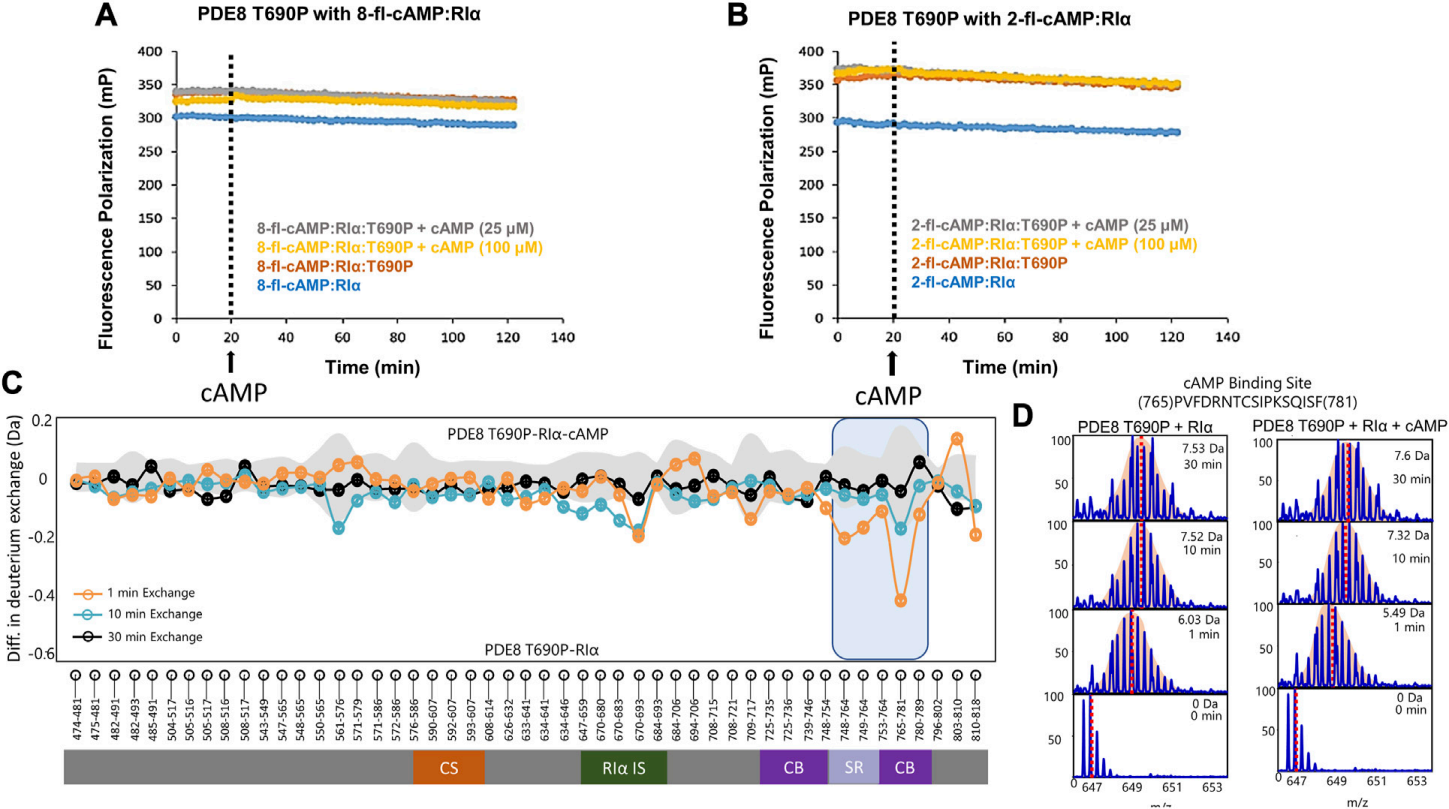
PDE8 T690P does not channel cAMP in RIα:PDE8 T690P complexes. Fluorescence polarization assays of RIα:PDE8 T690P complexes with **(A)** PDE non-hydrolyzable fluorescent 8-fl-cAMP and **(B)** PDE hydrolyzable 2’-fl-cAMP. cAMP (25, 100 μM) was added at t = 20 min as denoted by the dashed line. **(C)** Deuterium exchange difference (average number of deuterons) mapped for PDE8 T690P in RIα:PDE8 T690P in the presence of excess cAMP *versus* RIα:PDE8 T690P for peptic fragment peptides from N- to C- terminus. Negative differences denote decreased exchange (blue boxes) in the presence of excess cAMP. Domain organization of PDE is indicated: CS—catalytic site, RIα IS—RIα interaction site, CB—cAMP binding site, and SR—substrate recognition site. Standard deviations from replicate measurements are in gray. **(D)** Stacked mass spectra for cAMP binding site peptides 765–781. Left panel-mass spectral envelope of the indicated peptide in RIα:PDE8 T690P, right panel-mass spectral envelope of the indicated peptide in RIα:PDE8 T690P in the presence of excess cAMP. The deuterium uptake and deuteration time are shown for each spectra on the top right. Centroids are indicated by red dashed lines.

Threonine 690 on PDE8 spans the RIα interaction interface in the RIα:PDE8 complex (Krishnamurthy et al., 2014). We utilized FP to compare the interactions of RIα with PDE8 T690P with our previous WT RIα:PDE8 interaction studies (Tulsian et al., 2017; Tulsian et al., 2020; Tulsian et al., 2021). PDE8 T690P was complexed with RIα in the presence of two fluorophore-tagged analogs of cAMP—i) PDE8-hydrolyzable 2’-[fluo]-cAMP and ii) PDE8-non hydrolyzable 8-[fluo]-cAMP (Schaap et al., 1993) as described in methods. Larger polarization values were observed for RIα:PDE8 T690P complexes, relative to WT RIα for both 8-fl-cAMP- and 2′-fl-cAMP-bound RIα, across our experimental time course of 120 min, indicating stable complexation of RIα with PDE8 T690P (Figures 3A, B). The difference in FP (mP) between 2′-fl-cAMP and 8-fl-cAMP is attributable to the chemical differences between both fluorophores. The position of the fluorescein moiety is at the 2′OH in 2′-fl-cAMP and at the 8-position of the adenine group in 8-fl-cAMP, resulting in different rates of tumbling. This together with the altered excitation and emission wavelengths in the fluorophore accounts for differences in intrinsic polarization for the two fluorescent cAMP analogs. A similar magnitude FP readings for both WT RIα:PDE8 and RIα:PDE8 T690P in the presence of 8-fl-cAMP indicated that the T690P mutation did not impact complexation between RIα and PDE8 (Supplementary Figure S3).

### 3.2 T690P mutation abolishes competitive displacement of cyclic nucleotides in the RIα:PDE complex

Excess cAMP (25, 100 μM) was added to RIα:PDE8 T690P at t = 20 min to monitor competitive displacement of PDE-hydrolyzable and non-hydrolyzable fluorophore cAMP analogs as described previously (Tulsian et al., 2020). PDE-mediated hydrolysis of cAMP to 5′AMP resulted in rapid recovery of FP values to baseline as the product 5′AMP is competitively displaced by the fluorophores due to its lower affinity to RIα, relative to incoming cyclic nucleotides. Interestingly, addition of excess cAMP to WT 8-fl-cAMP-bound RIα:PDE showed a partial decrease in polarization compared to when cAMP was added to 8-fl-cAMP-bound RIα. The partial decrease corresponded to competitive displacement of 8-fl-cAMP from only two sites in the RIα–PDE complex (Supplementary Figure S3). No drops in FP values were observed in RIα:PDE8 T690P upon addition of excess cAMP, indicating an abolishment of competitive displacement of fluorescent cAMP/AMP from all four sites (Figures 3A, B). To determine if this was due to reduction in rates of cAMP hydrolysis in PDE8 T690P, we tested the ability of PDE8 T690P to hydrolyze bulk cAMP in solution and facilitate reassociation of the non-hydrolyzable fluorescent analog 8-Fl-cAMP into the RIα:PDE8 complex measured through addition of PDE8 T690P at t = 20 min (Supplementary Figure S3). A full recovery of FP to baseline value was observed at t = 40 min, indicative of complexation with RIα upon addition of PDE8 T690P. This would occur if PDE8 T690P was catalytically active, wherein product 5′AMP is displaced by non-hydrolyzable 8-fl-cAMP (Supplementary Figure S3). Since pre-formed T690P complexes do not release bound cAMP through competitive displacement by excess cAMP, recovery in FP upon PDE-mediated hydrolysis of cAMP indirectly reports hydrolysis of unbound cAMP by PDE8 T690P not bound to RIα.

PDE8 T690P showed reduced cAMP hydrolysis compared to WT PDE8 (Supplementary Figure S4A). For WT PDE8, a K_M_ of 1.13 ± 0.33 μM, V_max_ of 0.12 ± 0.01 μM/min (per nM of enzyme), and/k_cat_ of 2.01 ± 0.19 s^−1^ were calculated, which is similar to the values previously determined for free WT PDE8 (Yan et al., 2009; Tulsian et al., 2017). Correspondingly for PDE8 T690P, we obtained a K_M_ of 1.28 ± 0.38 μM, V_max_ of 0.09 ± 0.01 μM/min (per nM of enzyme), and a k_cat_ of 1.5 ± 0.16 s^−1^ (Supplementary Figure S4A). A ∼25% reduction in the k_cat_ observed in PDE8 T690P is indicative of an allosteric effect of the T690P mutation on the distal catalytic site of the PDE. These results indicate that PDE8 T690P shows lower activity, but RIα:PDE8 T690P is catalytically inactive and unresponsive to cAMP.

### 3.3 PDE8 T690P mediates asymmetric interactions with both CNBs of RIα

We applied HDXMS to map the effects of T690P on the conformational dynamics of the RIα:PDE8 complex. HDXMS analysis of RIα:PDE8 T690P yielded 49 peptides, with a total sequence coverage of 78.2% for PDE8 T690P (Supplementary Figure S5). Comparative HDXMS of RIα:PDE8 T690P and PDE8 T690P showed few differences in exchange. Only one peptide spanning the substrate binding site (peptides 765–781) showed marginal decreases in exchange upon RIα binding (0.44 ± 0.07 Da at t_ex_ = 1 min) (Supplementary Figure S6A).

HDXMS analysis of RIα yielded 33 peptides with 63.2% sequence coverage in RIα (Supplementary Figure S10) and was used to map the corresponding PDE8 T690P interface on RIα (Supplementary Figure S6B). Deuterium exchange protection (>0.5 Da) was observed only at the CNB:A interfacial site peptides 157–171 (0.7 ± 0.05 Da at t_ex_ = 30 min), while the CNB:B interfacial site peptides 271–290 (2.27 ± 0.05 Da at t_ex_ = 30 min) showed significantly increased deuterium exchange (>0.5 Da) in response to PDE8 T690P interactions. The magnitude of deuterium exchange protection at peptides 157–171 in CNB:A remained constant throughout the HDXMS time course (∼0.8 Da across 1–30 min), while the deuterium exchange in peptides 271–290 at CNB:B increased with deuteration time (0.62 Da at t_ex_ = 1 min to 2.26 Da at t_ex_ = 30 min). Significant increases in deuterium exchange (>0.5 Da) were also observed at the PBCs of both CNBs (peptides 204–221 in CNB:A and 328–336 in CNB:B), indicating release of cAMP from RIα to PDE8. These results are suggestive of each monomer in the PDE8 T690P dimer interacting asymmetrically with RIα. This is consistent with a model in which one monomer of the PDE dimer alone engages CNB:A of RIα (Doskeland and Ogreid, 1984; Guo and Zhou, 2016; Tulsian et al., 2020).

### 3.4 PDE8 T690P:RIα complexes are unresponsive to excess cAMP

HDXMS analysis comparing the dynamics of RIα:PDE8 T690P in the presence and absence of excess cAMP (330 μM) revealed no major differences in deuterium exchange, except for small magnitude deuterium exchange protection at a single locus at the substrate binding site peptides 765–781 (0.42 ± 0.05 Da at t_ex_ = 1 min) (Figures 3C, D). Absence of protection at peptides spanning the RIα binding interface such as peptides 670–693 (0.21 ± 0.03 Da) indicates that the addition of excess cAMP did not significantly enhance complexation between PDE8 T690P and RIα, which is consistent with our FP results (Figures 3A, B). No differences in deuterium exchange were seen at the corresponding interfaces in RIα (Supplementary Figure S8). Together, these results reveal that the RIα:PDE8 T690P complexes remained unresponsive to extraneous cAMP.

### 3.5 cAMP channeling is slower in RIα T207A:PDE8 complexes

Like Thr 690 of PDE8, Thr 207 in RIα is also positioned at the RIα:PDE8 interface in the signal termination complex (Krishnamurthy et al., 2014). Comparative HDXMS analysis of RIα T207A bound to PDE8 and RIα T207A was performed to map the impact of T207A on the RIα:PDE8 complex. We obtained a sequence coverage of 68.4% with 46 peptides for RIα T207A (Supplementary Figure S11). Significant increases in deuterium exchange (>0.5 Da) were observed at multiple loci at longer time points of deuterium exchange (t_ex_>10 min) in the RIα T207A:PDE8 complex, relative to free RIα T207A (Supplementary Figure S12). Peptides spanning both CNBs showed significant increases (>0.5 Da) in deuterium exchange with the CNB:A PBC peptides 202–221 showing the largest magnitude increase (1.6 ± 0.06 Da, t_ex_ = 30 min), while the CNB:B peptides 330–336 showed 0.55 ± 0.05 Da (t_ex_ = 30 min) increase in deuterium exchange (Supplementary Figure S12). This reflects translocation of endogenous cAMP from the PBC of RIα T207A to the active site of PDE8 (Tulsian et al., 2017). Increased dynamics were also observed at longer deuterium exchange time points (10, 30 min) at the PDE8 binding interface site on both CNBs (0.5 Da for peptides 157–172 in CNB:A and peptides 275–290 in CNB:B), suggestive of transient complexation between RIα T207A and PDE8 also found in WT RIα:PDE8 (Supplementary Figure S12) (Tulsian et al., 2017).

**FIGURE 4:**
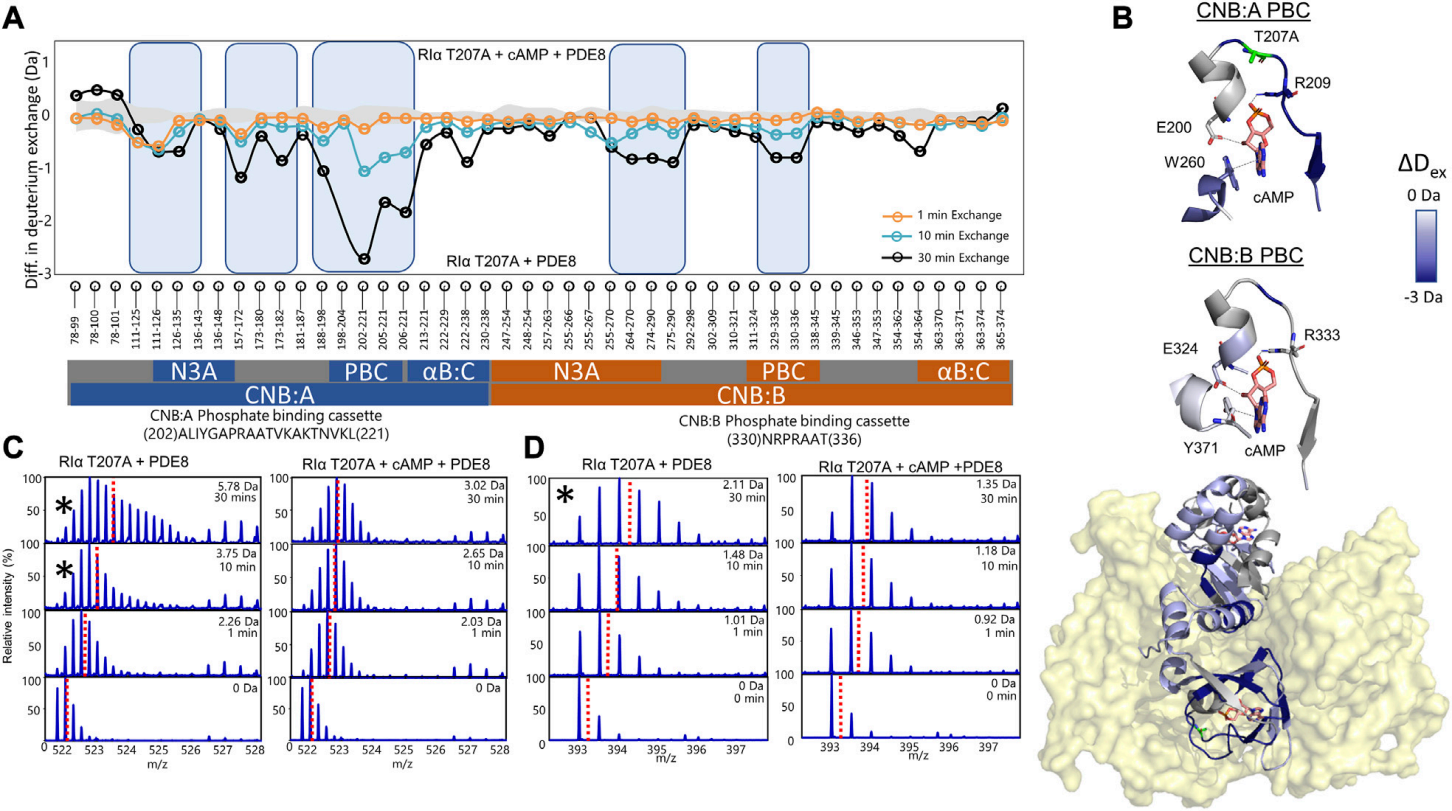
cAMP channeling is disrupted in RIα T207A:PDE8 complexes. **(A)** Deuterium exchange difference plot (excess cAMP) in RIα T207A:PDE8 for peptic fragment peptides from the N- to C- terminus (*X*-axis). Negative differences indicate decreased exchange (blue boxes) in the presence of excess cAMP. Standard deviations from replicate measurements are in gray. **(B)** Deuterium exchange differences (t_ex_ = 30 min) in excess cAMP mapped onto the docking model of RIα:PDE8. Top panels—closeup view of the phosphate binding cassettes of CNB:A and CNB:B with critical cAMP contacts in gray. **(C)** Stacked mass spectra for CNB:A peptides 202–221 and **(D)** CNB:B peptides 330–336. The left panel shows mass spectra of indicated peptide from RIα T207A:PDE8, right panel—mass spectra of RIα T207A:PDE8 in the presence of cAMP. Mass spectra demonstrating spectral broadening indicated by ‘*’ were analyzed by bimodal deconvolution. Centroids for the envelopes are indicated by red dashed lines.

Mass spectral broadening was observed in peptides spanning both CNB sites such as peptides 202–221 in CNB:A and peptides 330–336 in CNB:B of RIα T207A:PDE8, indicative of ensemble behavior. To determine if this ensemble behavior reflected two distinct conformational states of RIα, T207A—a low exchanging cAMP-bound population and high exchanging cAMP-free population, we performed bimodal deconvolution and analysis by HX-Express as described in Materials and Methods (Figure 4C). Bimodal spectra at CNB:A peptides were more clearly discernible and quantifiable at longer time points (t_ex_>10 min), unlike in WT RIα:PDE8 complexes where bimodal spectra for the same peptide were observed at earlier time points (t_ex_>30 s) (Tulsian et al., 2017). In the CNB:A reporter peptides 202–221, the cAMP-free population only represented 14.3% ± 3% of the overall spectra at t_ex_ = 10 min and 35% ± 1.7% at t_ex_ = 30 min (Supplementary Figure S13; Table 1). Based on this, the half-life of cAMP-release from RIα T207A in RIα T207A:PDE8 is estimated to be ∼45 min in contrast to WT complexes, where the lower exchanging population was completely absent by t_ex_ = 30 min (Tulsian et al., 2017). This indicated significantly (∼15-fold) slower rates of nucleotide channeling at CNB:A in RIα T207A:PDE8 complexes. In the CNB:B reporter peptides 330–336, spectral broadening indicative of ensemble behavior was only observed at t_ex_ = 30 min (Supplementary Figure S13; Table 1). The spectral broadening at CNB:B was unresolvable. These results validate our previous observation that cAMP release is a step-wise process where cAMP release occurs first from CNB:A followed by CNB:B (Doskeland and Ogreid, 1984; Guo and Zhou, 2016).

### 3.6 Excess cAMP is trapped in RIα T207A:PDE8 complexes

To assess the impact of T207A mutation on channeling of excess cAMP, comparative HDXMS analysis was performed for RIα T207A:PDE8 in the presence and absence of excess cAMP (330 μM) (Figure 4). Significant deuterium exchange protection (>0.5 Da) was observed at both CNB sites and the RIα:PDE8 interface in the complex in the presence of excess cAMP (Figures 4A, B). Deuterium exchange protection was observed throughout the time course of the reaction, indicating that the cyclic nucleotide was unable to translocate to the PDE8 active site through the cyclic nucleotide channel. Deuterium exchange protection was observed at CNB:A PBC peptides 202–221 (2.75 ± 0.05 Da, t_ex_ = 30 min), whereas CNB:B peptides 330–336 showed a smaller 0.8 Da protection (Figures 3C, D), attributable to the shorter CNB:B peptide. The average deuterium exchange/number of exchangeable amides for both CNBs was the same (∼0.15 Da). In excess cAMP, unimodal binomial spectra were observed in the CNB:A reporter peptides 202–221 and CNB:B peptides 330–336 throughout our experimental time-course of deuterium exchange (1–30 min), with a centroid value of the spectra comparable to the low-exchanging cAMP-bound population (Figure 4; Table 1).

To determine if 5′AMP channeling was disrupted to a similar extent by the T207A mutation, we carried out comparative HDXMS of RIα T207A:PDE8 in the presence of excess 5′AMP (3 mM) and RIα T207A:PDE8. Significant magnitudes of deuterium exchange protection (>0.5 Da) were observed at both CNB sites, which is comparable to the protection observed during cAMP channeling. This indicated that 5′AMP remained bound to RIα T207A in the RIα T207A:PDE8 complex and was not channeled out of RIα T207A (Supplementary Figure S14). Additional protection from deuterium exchange was observed in peptides spanning the B:C-helix. For instance, peptides 222–238 showed a protection of 1.73 ± 0.05 Da at t_ex_ = 30 min (Supplementary Figure S15). To determine if cAMP binding to RIα was also impaired by the T207A mutation, we performed comparative HDXMS analysis of RIα T207A with excess cAMP (330 μM) and RIα T207A bound to endogenous cAMP. Deuterium exchange protection was observed at both CNBs in excess cAMP, indicating that more cAMP was required to elicit deuterium exchange protection in RIα T207A compared to WT RIα (Supplementary Figure S15). This is indicative of weaker cAMP affinity in both CNBs in RIα T207A. Taken together, these results reveal that the T207A mutation on RIα disrupts nucleotide release from the CNB site of RIα during PDE hydrolysis in the RIα:PDE8 complex.

## 4 Discussion

**FIGURE 5:**
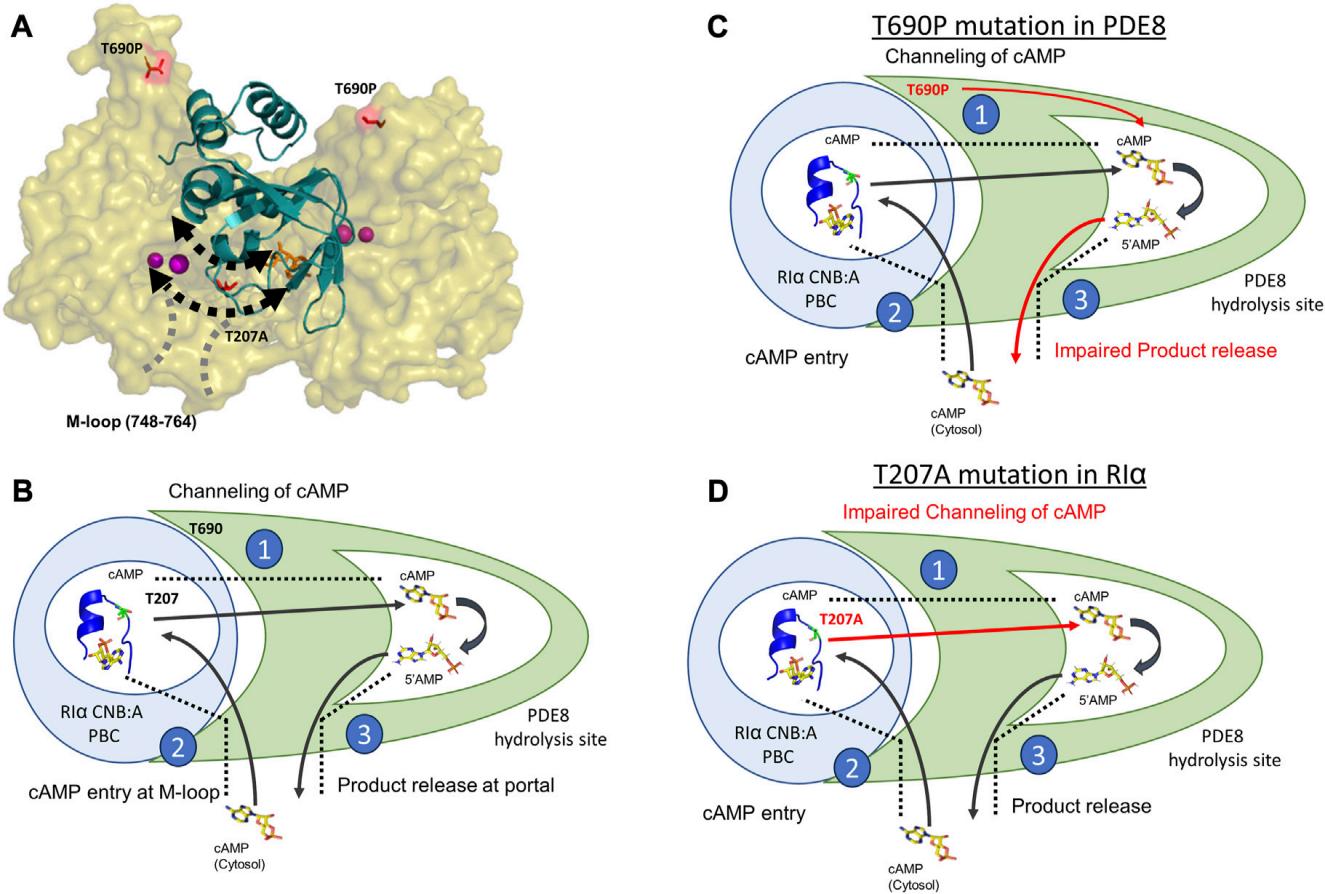
Molecular basis for disruption of hydrolysis by RIα T207A and PDE8 T690P **(A)** Docking model of RIα:PDE8 (Krishnamurthy et al., 2014). RIα CNB:A (cartoon) is shown in teal, and the PDE8 catalytic domain (surface) is shown in olive. cAMP (orange sticks) and metal ions within the PDE8 catalytic site (magenta). T690P and T207A mutation sites are shown as red sticks. The boundaries of the cAMP channel are represented by dashed black lines, where the arrows indicate channeling of nucleotides to the PDE8 active site. The M-loop (region corresponding 748–764 in PDE8) responsible for exchange of nucleotides between the cytosol and the RIα:PDE8 complex is represented by dashed gray lines **(B)** Model of the mechanism of cAMP hydrolysis in RIα:PDE8 complexes described by three steps: Step 1) Channeling: translocation of cAMP from the CNB of RIα to the PDE catalytic site for hydrolysis. Step 2) Processivity: binding of free cAMP from the cytosol at both CNBs of RIα. Step 3) Product (5′AMP) release from the PDE hydrolysis site through competitive displacement by incoming cAMP from the second hydrolysis cycle **(C)** and allosteric effect of T690P mutation on product release. **(D)** Effect of T207A mutation on cAMP channeling. The blue circle represents RIα CNB:A with the PBC enclosed inside the white circle and shown as a blue cartoon (PDE ID:1RGS). The T207/A207 site is colored in lemon green within the PBC and shown as sticks. The PDE8 catalytic domain is shown in green with the catalytic pocket/hydrolysis site enclosed in the white pocket within it. cAMP and 5′AMP are shown as sticks with arrows representing the movement of nucleotides. Red arrows highlight the step in the cAMP hydrolysis impacted by the mutations. The boundaries of the cAMP channel are shown as black dashed lines.

The process of cAMP channeling to the active site of PDE followed by hydrolysis to 5′AMP catalyzed by RIα:PDE complexes can be divided into the following three steps: Step 1) Channeling: translocation of cAMP from the CNB of RIα to the PDE catalytic site for hydrolysis. Step 2) Processivity: binding of free cAMP from the cytosol at both CNBs of RIα. Step 3) Product (5′AMP) release from the PDE hydrolysis site through competitive displacement by new cAMP from the cytosol (Figures 5A, B). Our results reveal that the two ACRDYS mutations in the PDE (T690P) and RIα (T207A) disrupted two distinct steps in processive cAMP hydrolysis in RIα:PDE complexes.

### 4.1 PDE8 T690P disrupts product 5′AMP release from the RIα:PDE8 complex

PDE8 T690P is homologous to PDE4D4 T594P, an established ACRDYS2 mutant (Supplementary Figure S1). However, unlike other ACRDYS2 mutants which demonstrate enhanced catalytic activity, PDE8 T690P demonstrated ∼25% lower catalytic rates, relative to WT PDE8. PDE8 WT and PDE8 T690P show similar K_McAMP_ ∼1.1 μM, indicating that both cAMP hydrolysis and product 5′AMP release are impacted by T690P mutation.

The crystal structure of the PDE8 catalytic domain (PDBID:3ECN) reveals that a proline substitution at the 690 position within a 3_10_ helix is likely to disrupt H-bonding, impacting multiple residues including K686, T690, L691, and E692 at the RIα interface (Wang et al., 2008; Morgan and Rubenstein, 2013). However, interactions between PDE8 T690P and RIα are maintained as demonstrated by FP and HDXMS (Figure 3; Supplementary Figure S6B). The lack of observable deuterium exchange differences on PDE8 T690P in response to complexation with RIα is attributable to asymmetric interactions, where only one monomer from dimeric PDE8 T690P interacts with both CNB:A sites across both chains of RIα (Supplementary Figure S6B). This indicates that the second monomer likely remains unbound and hence does not mediate interactions with CNB:B of RIα. This explains the lack of deuterium exchange differences on PDE8 T690P in response to complexation with RIα. This is consistent with the observation that CNB:A is the primary site of complexation with PDE8 and suggests a stepwise release of cAMP from each of the CNBs of RIα, where release of cAMP from CNB:A cooperatively promotes the release of cAMP from the tandem CNB:B site (Doskeland and Ogreid, 1984; Guo and Zhou, 2016; Tulsian et al., 2020).

Processive cAMP hydrolysis was severely impaired in RIα:PDE8 T690P, and no changes in FP or HDXMS were observed upon addition of excess cAMP (Figure 3; Supplementary Figure S3). This indicated that while RIα:PDE8 T690P underwent one cycle of channeling and hydrolysis, the RIα:PDE8 T690P complex remained in an inactive state after just one round of cAMP channeling and hydrolysis. This disrupted its ability to catalyze processive hydrolysis of cAMP. In contrast, WT RIα:PDE8 showed full processivity of cAMP hydrolysis, which was captured by competitive displacement by excess cAMP of both fluorescent analogs (Supplementary Figure S3) (Krishnamurthy et al., 2014; Tulsian et al., 2017; Tulsian et al., 2020). HDXMS showed no differences in dynamics between RIα:PDE8 T690P and PDE8 T690P at key catalytic loci including the substrate binding site (peptides 725–746) and catalytic active site (peptides 590–607) (Figures 4C, D) (Tulsian et al., 2017). Deuterium exchange protection observed at the substrate-binding site (765–781) in PDE8 T690P at earlier time points (t_ex_ = 1 min) indicates ligand/product nucleotide retention at the PDE8 catalytic site before being released. Since PDE8 T690P is catalytically active, the protection at this site is attributable to product 5′AMP remaining bound to PDE8 T690P.

The substrate recognition site (748–765) spans the nucleotide channel in PDE8 and likely plays a role in product release from the nucleotide channel. This is inferred from the enhanced dynamics in this region, indicative of conformational changes during movement of nucleotides (Tulsian et al., 2017; Tulsian et al., 2020). In contrast, this region did not show differences in deuterium exchange in T690P complexes, indicating that no conformational changes in this region. We infer that this site functions both as a portal for processive cAMP recruitment from bulk cytosol into the RIα:PDE8 active sites and for release of 5′AMP. Closure of this region during channeling signified by the absence of deuterium exchange differences in T690 complexes is indicative of trapping of product 5′AMP within the complex and is consistent with our FP results (Figures 3A, B). Trapping 5′AMP within the PDE active site in T690P complexes would disrupt processive cAMP channeling from RIα and trap cAMP within RIα:PDE8. This ‘backing up’ would eventually arrest processivity, wherein subsequent cycles of hydrolysis cannot be completed by the T690P signal termination complex (Figure 5C). Overall, T690P disrupts functioning of an allosteric network coupling the RIα interface to the substrate recognition site in RIα:PDE8. This allostery forms the basis for enhanced PDE catalysis in RIα:PDE8 complexes, relative to free PDE8 (Tulsian et al., 2017).

### 4.2 RIα T207A impairs processivity of cAMP hydrolysis through disruption of nucleotide channeling

Thr 207 is highly conserved in the PBC of CNBA but does not mediate orthosteric interactions with cAMP (Su et al., 1995; Canaves et al., 2000). HDXMS analysis revealed that while cAMP channeling of RIα T207A:PDE8 complexes was slower, the process overall closely resembled that for WT RIα:PDE8 complexes (Tulsian et al., 2017). The delayed emergence of spectral broadening in RIα T207A:PDE8 complexes at t_ex_>10 min relative to WT RIα:PDE8 complexes (t_ex_ = 30 s) indicates a 15-fold slower channeling of cAMP from PBC (Figures 4C, D; Supplementary Figure S13) (Tulsian et al., 2017).

Addition of excess cAMP to track processive cAMP channeling and hydrolysis resulted in deuterium exchange protection (>0.5 Da) at both CNB sites, with the CNB:A domain conferring the largest magnitude of protection (2.8 Da at t_ex_ = 30 min) (Figures 3A, B). Furthermore, binomial spectra were observed at both CNBs, and the centroid values at CNB:A peptides were comparable to the calculated centroids for the lower-exchanging population in the RIα T207A:PDE8 complex (Figures 4C, D). The magnitude deuterium exchange protection at both PBCs A and B increased through the time course of the deuterium exchange experiment (t_ex_ = 1–30 min), revealing that the nucleotide was not released from both CNB sites of RIα T207A.

We have previously described that the PBC undergoes conformational changes upon PDE8 binding that weakens cAMP affinity at the PBC, leading to channeling (Tulsian et al., 2017). Thr 207 in RIα:PDE8 is critical for cAMP binding and release. A docking model of the RIα:PDE8 complex reveals positioning of Thr 207 at the cAMP channel interface between the CNB:A PBC and PDE8 hydrolysis site (Figure 5A) (Krishnamurthy et al., 2014). Interactions of the side chain of T207 with the phosphate moiety of cAMP or 5′AMP in RIα:PDE8 likely plays a critical role in channeling of cAMP out of the CNB pocket to the PDE8 hydrolysis site. This explains the slower channeling of cAMP observed in RIα T207A:PDE8 complexes. Addition of excess cAMP exacerbates the effect of this mutation since RIα possesses high affinity for cAMP. Since translocation of cAMP from the CNB of RIα is the first step in cAMP hydrolysis by signal termination, impairment of this step by the T207A mutation results in subsequent disruption to all downstream steps. Overall, this mutation greatly reduces cAMP channeling, which consequently impairs the processivity of cAMP hydrolysis from the bulk cytosol (Figure 5D).

### 4.3 Active site remodeling in RIα:PDE8 complexes

Our results highlight the extensive conformational changes in both the cAMP binding pocket on RIα and the active catalysis site on PDE8 upon complex formation. This explains how mutations on two interacting proteins impact cAMP processivity mediated through a nucleotide channel. Both RIα T207A and PDE8 T690P trap two conformational intermediates in the signal termination phase of PKA. This leads to impairment of cAMP processivity across multiple activation and termination cycles of PKA signaling. While T690P partially reduced the activity of PDE8, the activity of PKA C-subunit is not affected by any of these mutations. This reveals that the mutations disrupt the cAMP processivity that drives multiple rounds of activation and PKA reset. This results in higher concentrations of cAMP required for processive cycles of activation and termination. Adaptation to cAMP levels during multiple rounds of PKA activation and termination is an essential feature of PKA signaling. Our results reveal that specific mutations in PDEs and PKA RIα result in dysregulation of the adaptation response. This generates a hormonal hyperstimulation of GPCRs, characteristic of acrodysostosis (Figure 5).

A detailed picture of the conformational changes accompanying RIα:PDE8 complex formation will have to await high-resolution structures. The remodeled active site in these complexes offers a novel site for targeted inhibitor design. In summary, we have derived the impact of two ACRDYS-causing mutations on the signal termination phase of PKA. Both mutants demonstrate severe impairments in processive hydrolysis that render RIα:PDE8 unresponsive to fluxes in cAMP levels during multiple cycles of cAMP activation and termination. This impairment of processivity would result in unregulated PKA kinase activity and delayed reset of PKA to a basal inactive state. These results confirm that the pathophysiology of ACRDYS is contributed by higher thresholds for activation and termination phases of PKA.

## Data Availability

All mass spectrometry data has been deposited to ProteomeXchange PRIDE repository with the accession number PXD045088.
