# Supplementary figures and images for: Impaired cAMP processivity by phosphodiesterase-protein kinase A complexes in acrodysostosis
Source: Front Mol Biosci. 2023 Sep 21;10:1202268. doi: 10.3389/fmolb.2023.1202268 (PMC10552185; doi:10.3389/fmolb.2023.1202268)

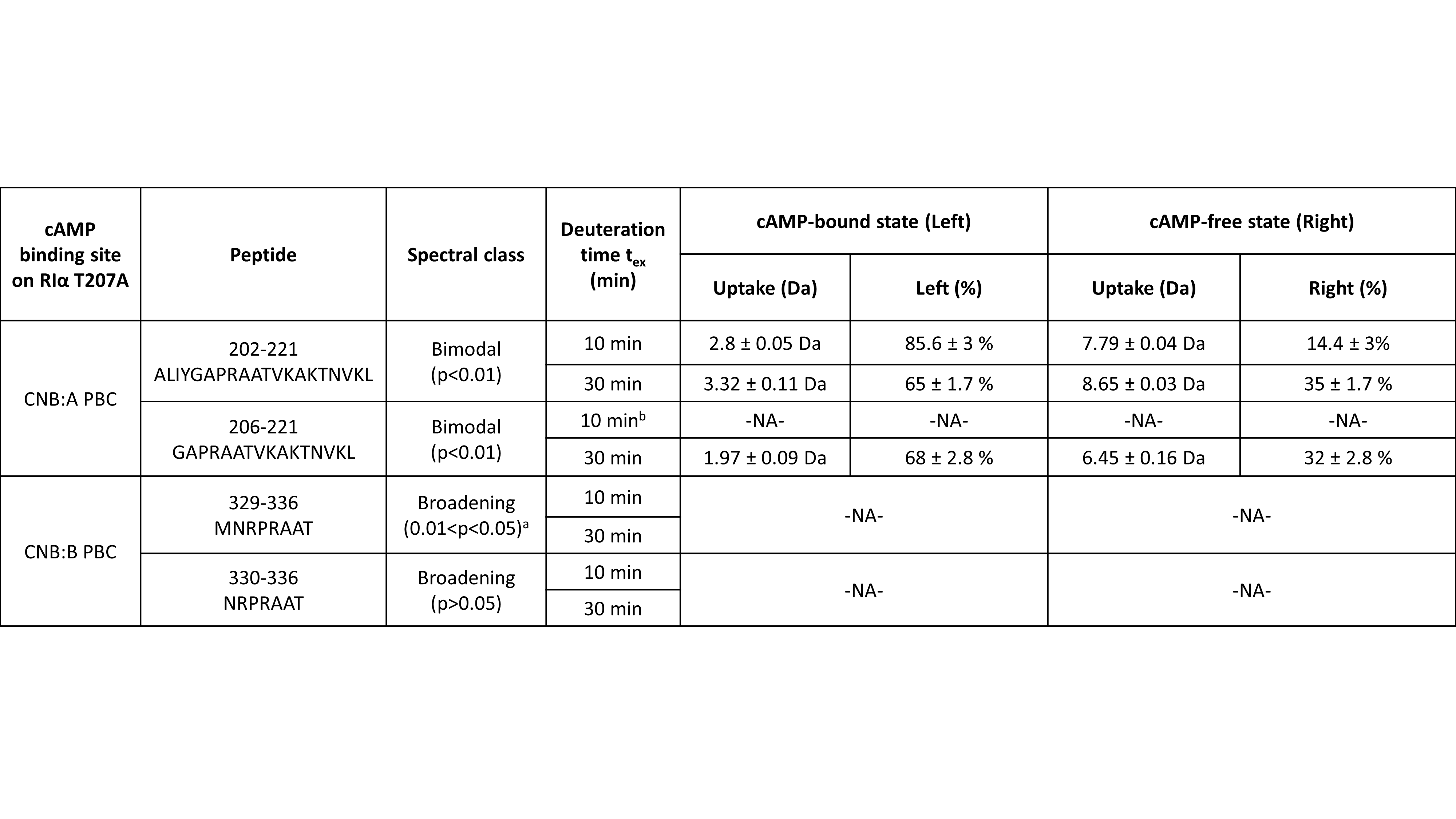

Supplement: Supplementary file 3 [file Image1.TIF]
