# Supplementary material for: Impaired cAMP processivity by phosphodiesterase-protein kinase A complexes in acrodysostosis
Source: Front Mol Biosci. 2023 Sep 21;10:1202268. doi: 10.3389/fmolb.2023.1202268 (PMC10552185; doi:10.3389/fmolb.2023.1202268)
Supplement: Supplementary file 4 [file DataSheet1.PDF]

Supplementary Material

Impaired cAMP Processivity by Phosphodiesterase-Protein Kinase A complexes in Acrodysostosis

Varun Venkatakrishnan[1]†, Abhijeet Ghode[2]†, Nikhil Tulsian[2], Ganesh Anand[1]*

*** Correspondence:** Ganesh Srinivasan Anand: gsa5089@psu.edu

† Contributed equally to the research and are first authors

1.1 Supplementary Figures

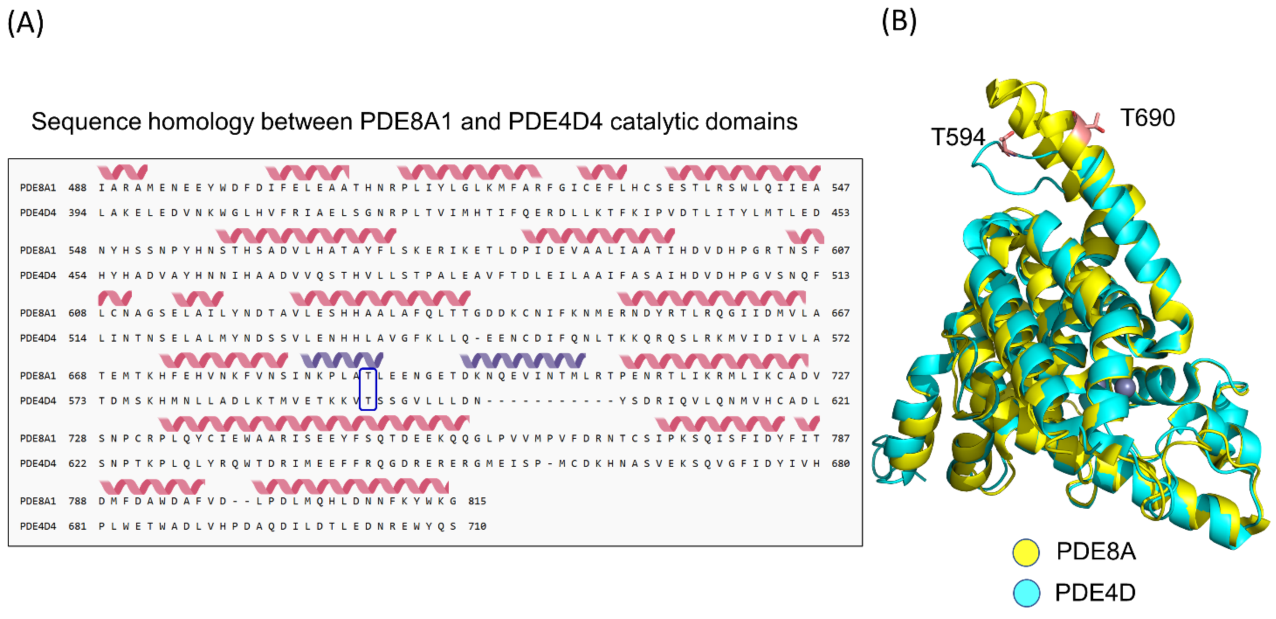

Supplementary figure 1: Structure and Sequence homology between PDE8A1 and PDE4D4: (A) Sequence homology between PDE8A1 (480-820) and PDE4D (380-715) catalytic domains with 68% sequence similarity from BLAST. The conserved mutation site T690 in PDE8A1 or T594P in PDE4D is highlighted in the blue box. Red colored helix-alpha helix, purple colored helix-3_{10} helix. **(B)** Structural homology between PDE4D4 and PDE8A1. Structural alignment of PDE4D (PDBID:1PTW) in cyan and PDE8A1 (PDBID:3ECM) in yellow with RMSD of 3.55Å. Mutation site T690 in PDE8A1/ T594 in PDE4D4 is highlighted in salmon.

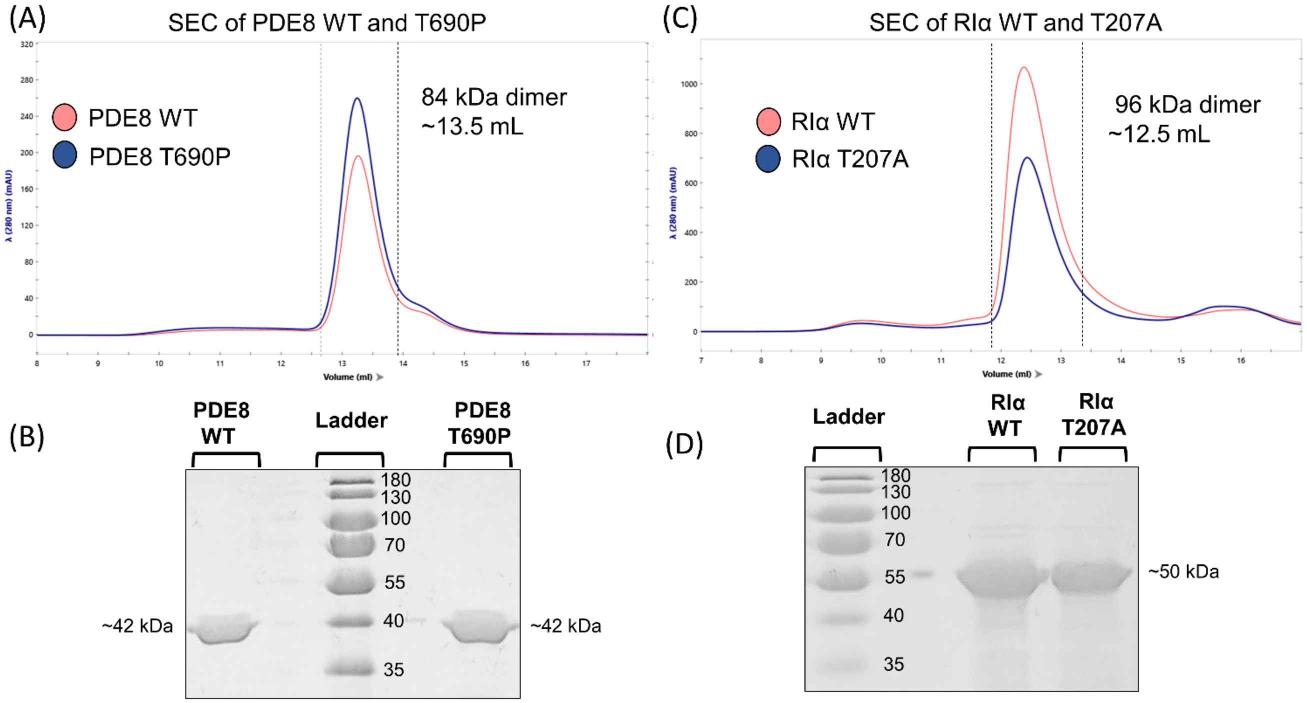

Supplementary figure 2: Recombinant expression and purification of proteins. (A) Size exclusion chromatogram for Re-folded catalytic domains of PDE8 WT (Pink trace) and PDE8 T690P (Blue trace). **(B)** Sodium dodecyl sulfate-polyacrylamide gel electrophoresis (SDS-PAGE) of Anion-exchange chromatography (AEC) eluted PDE8 WT (left lane), Page ruler pre-stained protein ladder (center lane) and PDE8 T690P (Right). **(C)** Size exclusion chromatogram of RIα WT (Pink trace) and RIα T207A (Blue trace) in an Enrich 650 SEC column (24 mL column volume). **(D)** SDS-PAGE of Ladder (left), Histag purified RIα WT (middle) and RIα T207A (right).

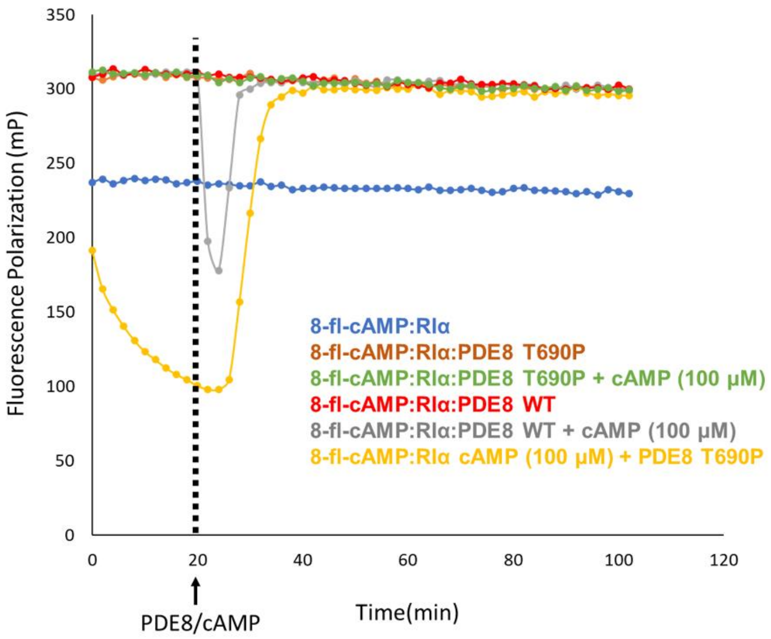

Supplementary figure 3: Fluorescence polarization with 8-fl-cAMP comparing hydrolysis and complexation for wild-type PDE8 and PDE8 T690P with RIα. PDE8/cAMP was added at 20 min.

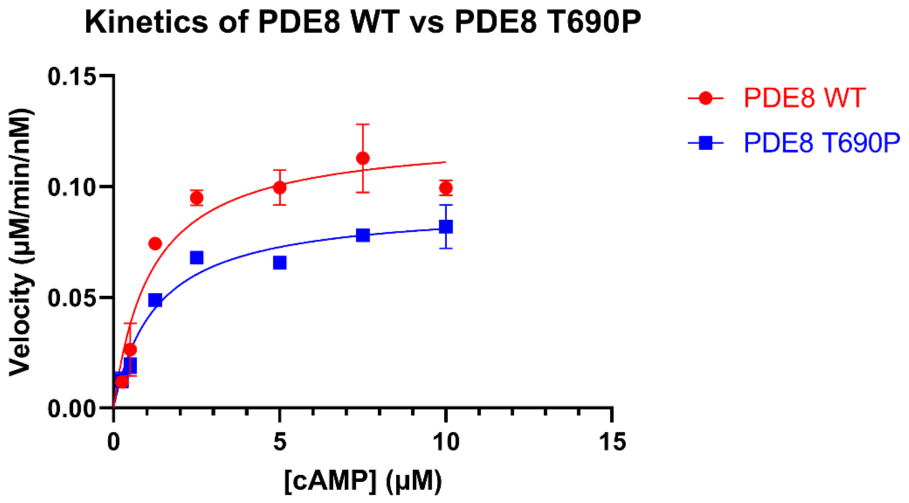

Supplementary figure 4: Impairment of cAMP hydrolysis in PDE8 T690P. Kinetics of cAMP hydrolysis by PDE8 WT (red trace) and PDE8 T690P (blue trace) as determined by BIOMOL green PDE assay. Measurements were taken in triplicates and error bars are shown for each data point. X-axis corresponds to substrate concentration in µM while Y-axis corresponds to reaction velocity representing µM of 5'AMP generated/min/nM of enzyme.

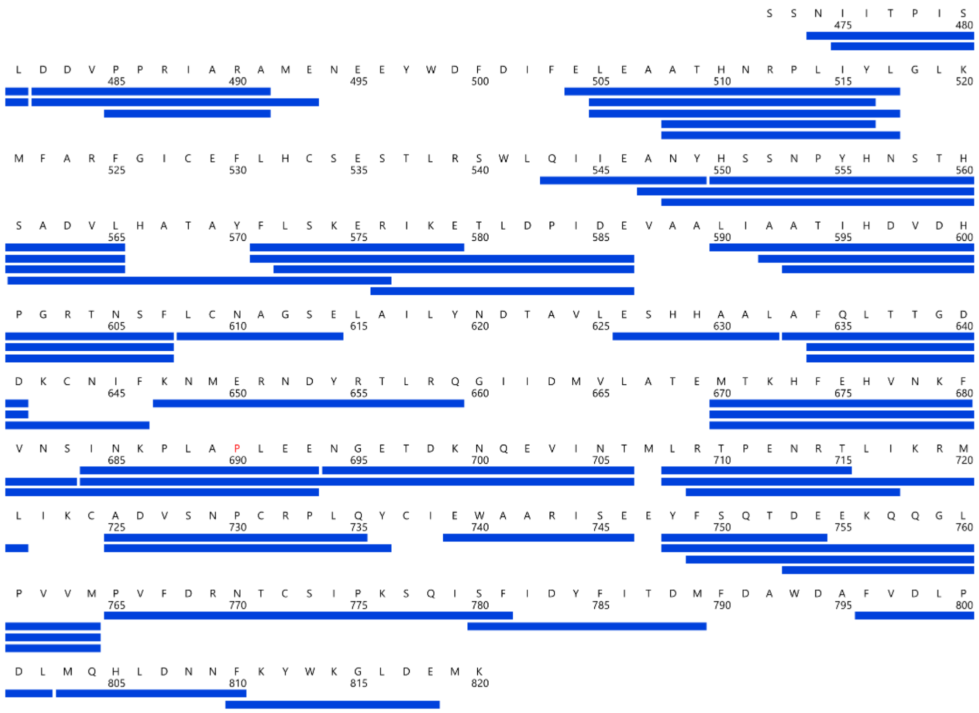

Total: 49 Peptides, 78.2% Coverage, 2.18 Redundancy

Supplementary figure 5: Sequence coverage map of PDE8 T690P. The mutation site T690P is highlighted in red. Total sequence coverage obtained is 78.2% with 49 peptides.

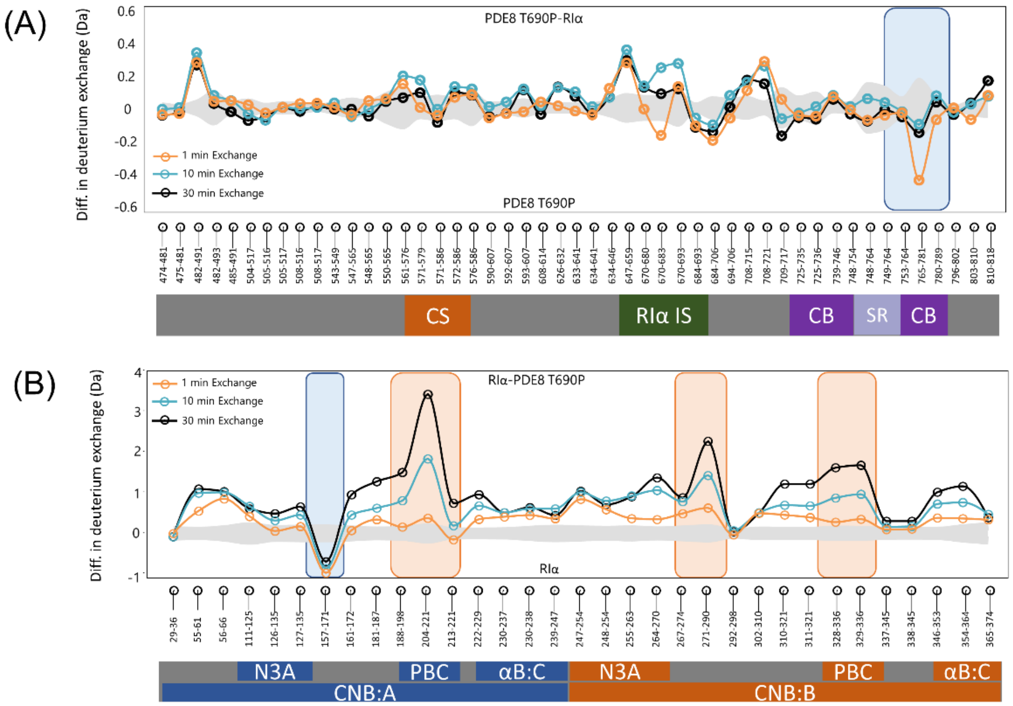

Supplementary figure 6: Altered interactions of PDE8 T690P with RIα. (A) Deuterium exchange difference in average number of deuterons exchanged in PDE8 T690P side for RIα:PDE8 T690P versus PDE8 T690P for peptic peptides from N- to C- terminus. Negative differences indicate decreased exchange (blue boxes) and positive differences indicate increased exchange (orange boxes) in response to RIα binding. Peptide labels are given for each peptide from the N-terminal to the C-terminal with the domain organization of PDE8 T690P given below. CS-catalytic site, RIα IS- RIα interaction site, CB-cAMP binding site and SR-substrate recognition site. Replicate errors are highlighted in gray. **(B)** Deuterium exchange difference in average number of deuterons exchanged in RIα side for RIα:PDE8 T690P versus RIα for peptic peptides from N- to C- terminus. Negative differences indicate decreased exchange (blue boxes) and positive differences indicate increased exchange (orange boxes) in response to PDE8 T690P binding. Peptide labels are given for each peptide from the N-terminal to the C-terminal with the domain organization of RIα given below. CNB:A/B-cyclic nucleotide binding domain, PBC-phosphate binding cassette, N3A,B:C-helices.Replicate errors are highlighted in gray.

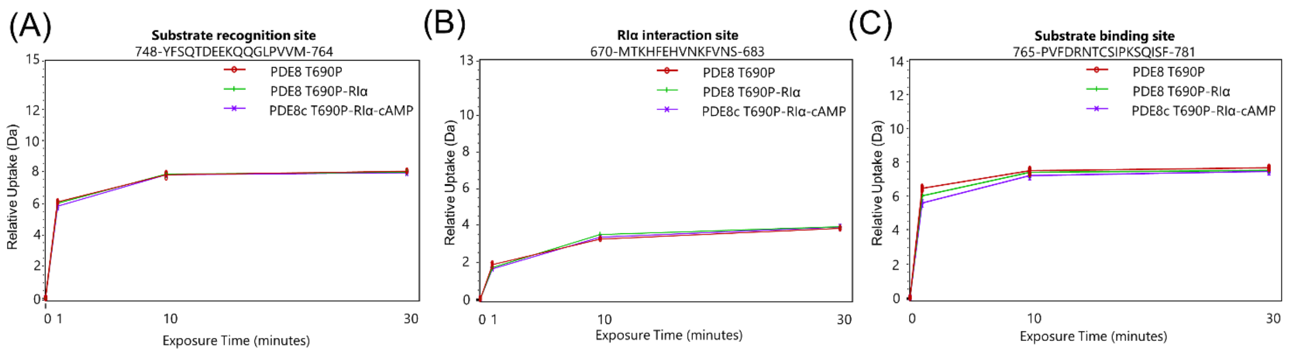

Supplementary figure 7: Deuterium exchange uptake plots for peptides in PDE8 T690P (A) Peptide 748-764 spanning the cAMP substrate recognition site **(B)** Peptide 670-683 spanning the RIα interaction site **(C)** Peptide 765-781 corresponding to the cAMP substrate binding site.

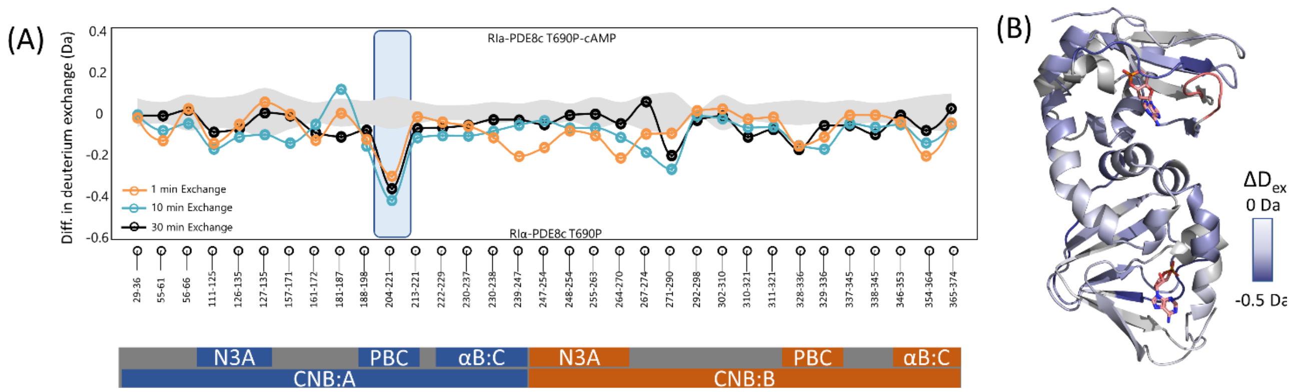

Supplementary figure 8: Processive hydrolysis is impaired in RIα:PDE8 T690P complexes. (A) Deuterium exchange difference in average number of deuterons exchanged in RIα RIα side in RIα:PDE8 T690P in the presence of cAMP versus RIα :PDE8 T690P for peptic peptides from N- to C- terminus. Negative differences indicate decreased exchange (blue boxes) in response to cAMP binding. CNB:A/B-cyclic nucleotide binding domain, PBC-phosphate binding cassette, N3A,B:C-helices. Replicate errors are highlighted in gray. **(B)** Heatmap representing deuterium exchange differences in RIα side in response to interactions with PDE8 T690P in the presence of excess cAMP. (PDBID:1rgs) at t=30 min. Deeper shades of blue correspond to deuterium exchange protection as given in key. The T207A mutation site is highlighted in green. Bottom-Zoomed-in view of the phosphate binding cassettes of CNB:A and CNB:B with orthosteric cAMP contacts in gray.

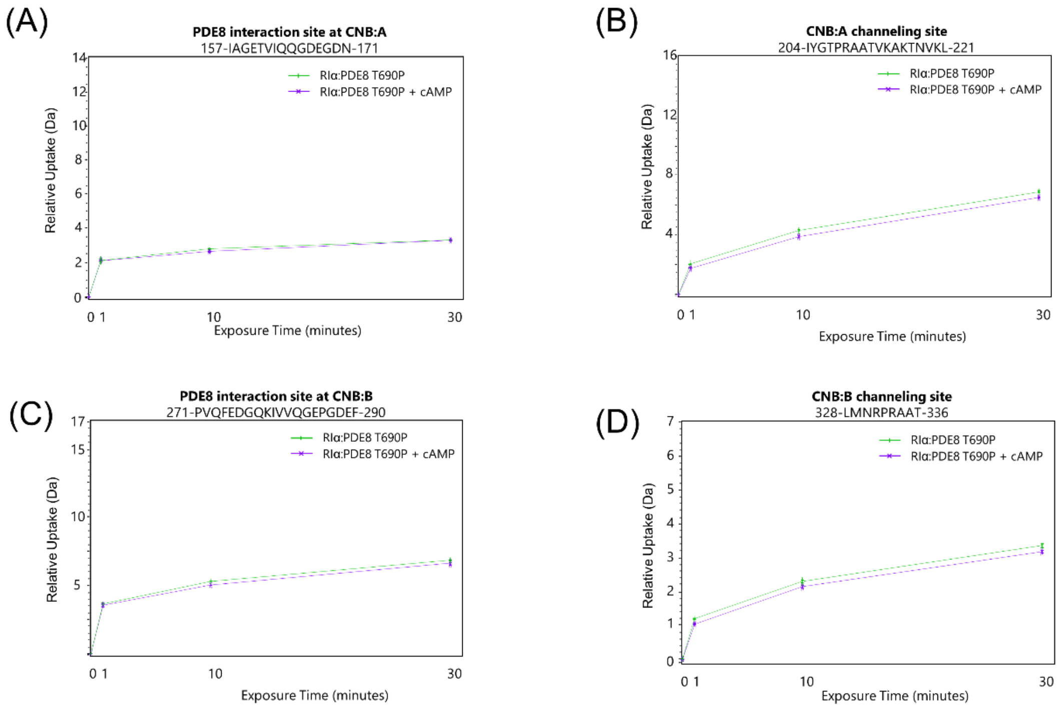

Supplementary figure 9: Deuterium exchange uptake plots for peptides in RIα during interactions with PDE8 T690P. **(A)** Peptide 158-172 spanning the PDE8 interaction site at CNB:A. (B)Peptide 203-222 spanning cAMP binding and channeling site at CNB:A **(C)** Peptide 272-291 spanning the PDE8 interaction site at CNB:B **(D)** Peptide 329-337 spanning cAMP binding and channeling site at CNB:B

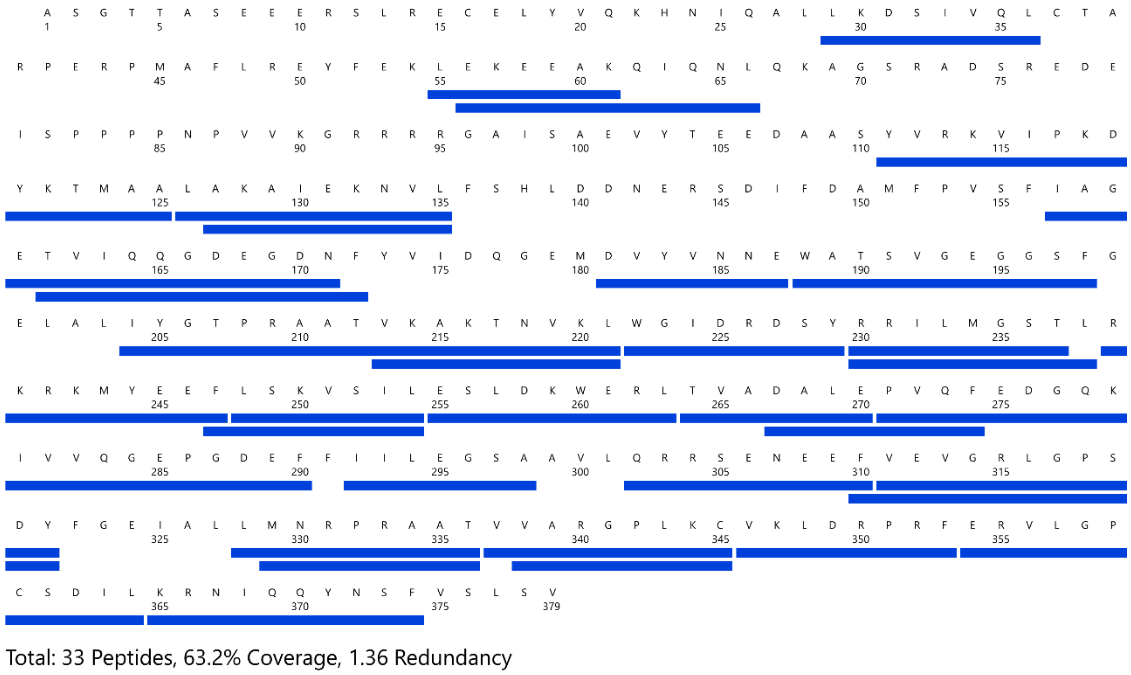

Supplementary figure 10: Sequence coverage map of RIα for PDE8 T690P: RIα interactions. Total sequence coverage obtained is 63.2% with 33 peptides.

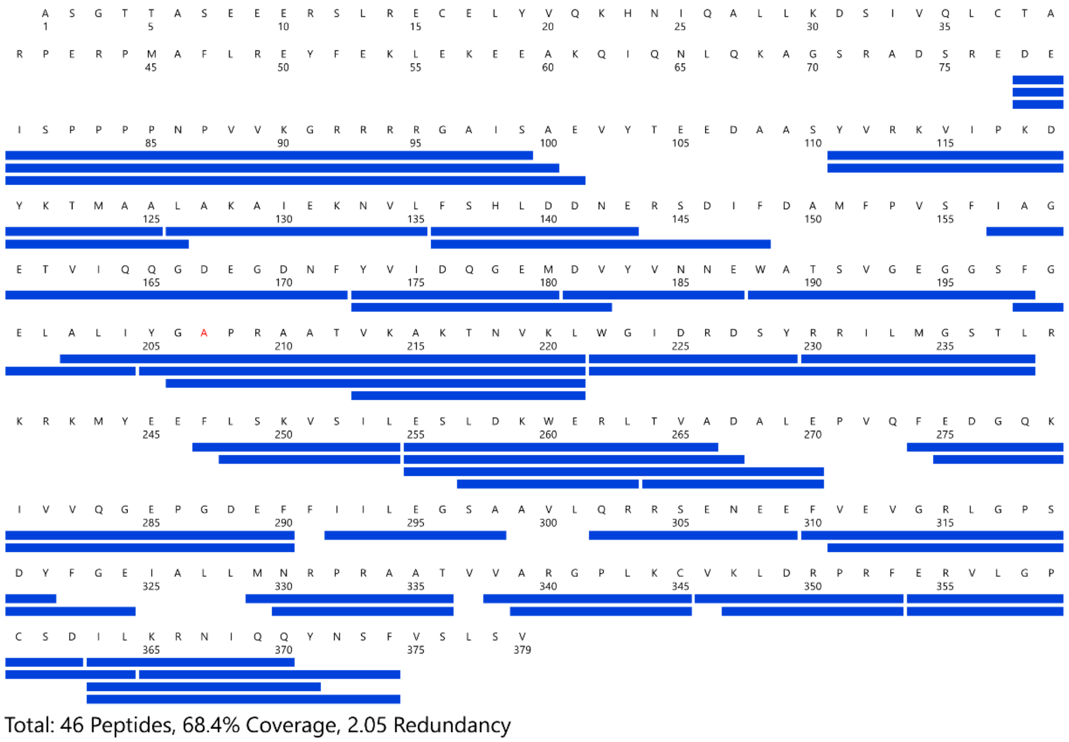

Supplementary figure 11: Sequence coverage map of RIα T207A. The mutation site T207A is highlighted in red. Total sequence coverage obtained is 68.4% with 46 peptides.

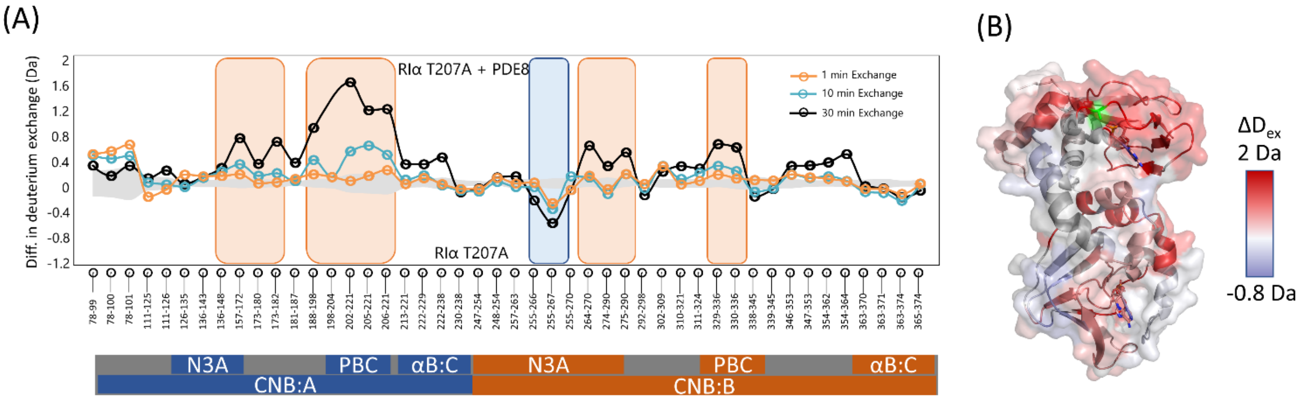

Supplementary figure 12: Channeling of cAMP in RIα T207:PDE8. (A) Deuterium exchange difference in average number of deuterons exchanged in RIα side for RIα T207A:PDE8 versus RIα T207A for peptic peptides from N- to C- terminus. Negative differences indicate decreased exchange (blue boxes) and positive differences indicate increased exchange in response to PDE8 binding. Peptide labels are given for each peptide from the N-terminal to the C-terminal with the domain organization of RIα T207A given below. CNB:A/B-cyclic nucleotide binding domain, PBC-phosphate binding cassette, N3A,B:C-helices. Replicate errors are highlighted in gray. **(B)** Heatmap representing deuterium exchange differences in RIα T207A complex in response excess cAMP (PDBID:1rgs) at t=30 min. Deeper shades of red correspond to increased deuterium exchange and shades of blue correspond to deuterium exchange protection as given in key. The T207A mutation site is highlighted in green.

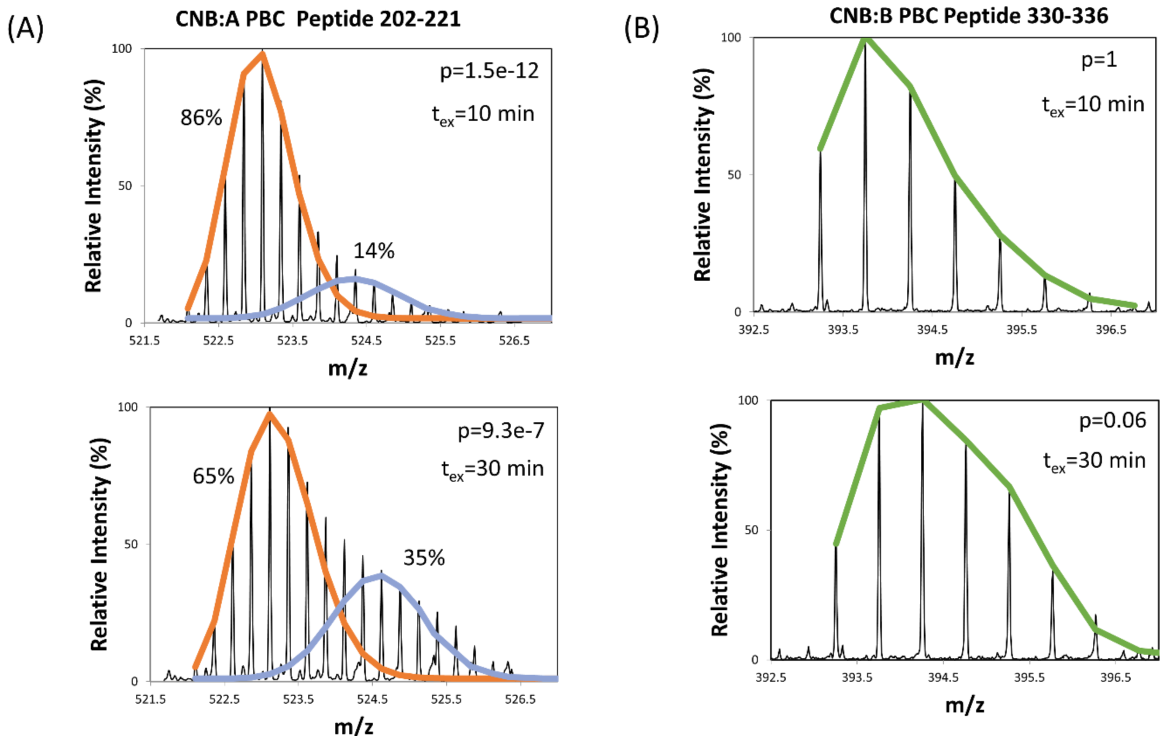

Supplementary figure 13: Monitoring cAMP release from CNBs in RIα T207A:PDE8 complexes. Bimodal deconvolution for **(A)** CNB:A PBC peptide 202-221 and **(B)** CNB:B PBC peptide 330-336. Top panels,t_{ex}=10 min, bottom panels, t_{ex}= 30 min. Orange spectral trace-low exchanging cAMP-bound population, blue trace-high exchanging cAMP-free population and green trace-spectral broadening.

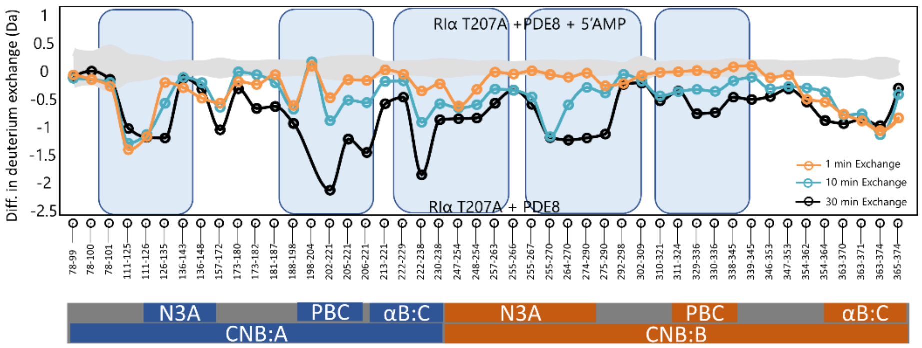

Supplementary figure 14: 5'AMP channeling in RIα T207A:PDE8 complexes. Deuterium exchange difference plot (excess 5'AMP) in RIα T207A:PDE8 for peptic fragment peptides from N- to C- terminus (X-axis). Negative differences indicate decreased exchange (blue boxes) in presence of excess 5'AMP. Replicate measurement errors are in gray.

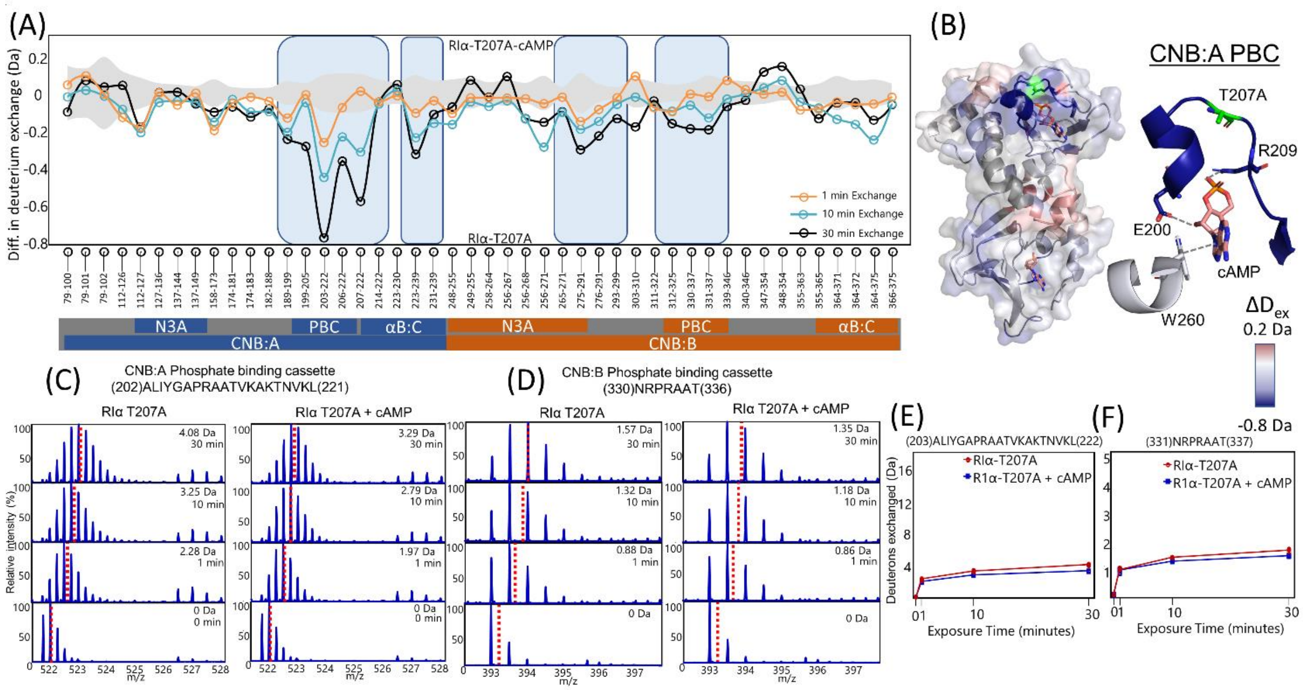

Supplementary figure 15: T207A mutation on RIα does not impair cAMP binding. (A) Deuterium exchange difference in average number of deuterons exchanged in RIα T207A in the presence of cAMP versus RIα T207A for peptic peptides from N- to C- terminus. Negative differences indicate decreased exchange (blue boxes) in response to cAMP binding. Peptide labels are given for each peptide from the N-terminal to the C-terminal with the domain organization of RIα T207A given below. CNB:A/B-cyclic nucleotide binding domain, PBC-phosphate binding cassette, N3A,B:C-helices. Replicate errors are highlighted in gray. **(B)** Left-Heatmap representing deuterium exchange differences in response to cAMP binding to RIα T207A (PDBID:1rgs) at t=30 min. Deeper shades of blue correspond to deuterium exchange protection whereas deeper shades of salmon correspond to increased deuterium exchange respectively as given in key. The T207A mutation site is highlighted in green. Right-Zoomed-in view of the phosphate binding cassette of CNB:A with orthosteric cAMP contacts in gray. **(C)** Mass spectra for CNB:A peptide 202-221 and (D) CNB:B peptide 330-33. Left-mass spectra of RIα T207A, right-mass spectra of RIα T207A in the presence of cAMP. (E) Deuterium exchange uptake plots for peptide 203-222 and (F) peptide 331-337 respectively comparing deuterium uptake (x-axis) as a function of exchange time (min) for RIα T207+cAMP (blue trace) vs RIα T207A (red trace).

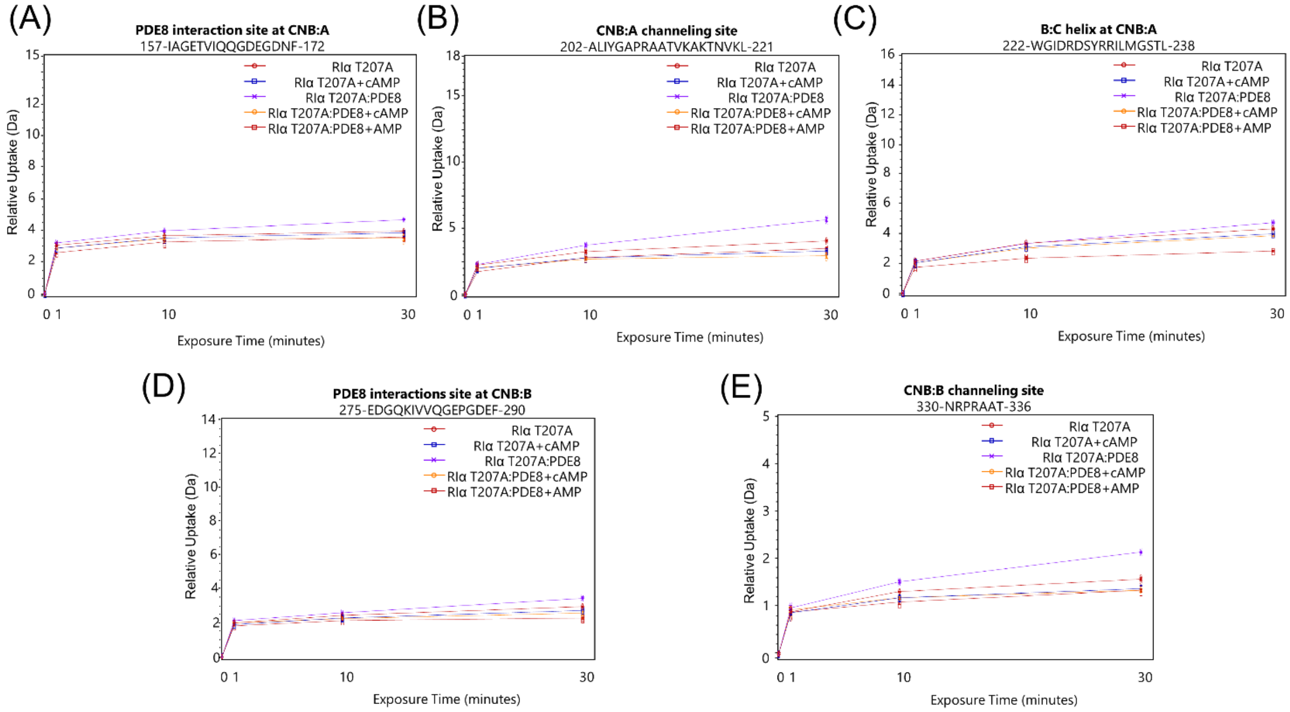

Supplementary figure 16: Deuterium exchange uptake plots for peptides in RIα T207A. (A) Peptide 157-172 spanning the PDE8 interaction site at CNB:A. **(B)**Peptide 202-221 spanning cAMP binding and channeling site at CNB:A **(C)** Peptide 222-238 in B:C helix of CNB:A**(D**) Peptide 275-290 spanning the PDE8 interaction site at CNB:B **(E)** Peptide 330-336 spanning cAMP binding and channeling site at CNB:B

1.2 Supplementary Tables

**Dataset**	**PDE8 T690P:RIα vs PDE8 T690P**		**PDE8 T690P:RIα + cAMP vs PDE8 T690P:RIα**		**RIα T207A:PDE8 vs RIα T207A**	**RIα T207A:PDE8+cAMP vs RIα T207A:PDE8**	**RIα T207A:PDE8+5'AMP vs RIα T207A:PDE8**	**RIα T207A+cAMP vs RIα T207A**
	**PDE8 T690P side**	**RIα side**	**PDE8 T690P side**	**RIα side**				
**Complexation stoichiometries**	PDE8 T690P complexed to RIα at 2:1 ratio				RIα T207A complexed to PDE8 at 2:1 ratio			No complexation with PDE8
**HDX reaction details**	3 μL sample (10 μM) + 27 μL deuterium exchange buffer (20 mM Tris-Cl, 50 mM NaCl, 5 mM $MgCl_2$ + 20 μM $ZnSO_4$ in 99.9% D_2O). Final deuteration – 89.99%. All deuterium exchange carried out at 25°C. Reactions quenched with 20 μL 0.1% Trifluoroacetic acid to bring pH to 2.5 For reactions with nucleotides, 330 μM cAMP and 3 mM 5'AMP was added to deuterium exchange buffer.							
**HDX time course**	0, 1, 10 and 30 min							
**Replicates**	3 Technical replicates							
**Number of peptides**	49	33	49	33	46			
**Sequence coverage**	78.2%	63.2%	78.2%	63.2%	68.4%			
**Peptide redundancy**	2.18	1.96	2.18	1.96	2.05			
**Significant difference threshold**	0.5 Da							

Supplementary table 1: Summary of HDXMS conditions

Sno.	Peptide	Start	End	Max Uptake (Deuterons)	MH+	Z	Number of deuterons exchanged at 1 min						Number of deuterons exchanged at 10 min						Number of deuterons exchanged at 30 min					
							PDE8 T690P		PDE8 T690P + RIα		PDE8 T690P + RIα + cAMP		PDE8 T690P		PDE8 T690P + RIα		PDE8 T690P + RIα + cAMP		PDE8 T690P		PDE8 T690P + RIα		PDE8 T690P + RIα + cAMP	
							Uptake	SD	Uptake	SD	Uptake	SD	Uptake	SD	Uptake	SD	Uptake	SD	Uptake	SD	Uptake	SD	Uptake	SD
1	NIITPISL	474	481	6	870.5295	1	3.99	0.02	3.95	0.02	3.94	0.03	3.95	0.03	3.95	0.02	3.94	0.02	4.00	0.03	3.96	0.02	3.94	0.02
2	IITPISL	475	481	5	756.4866	1	3.25	0.01	3.22	0.02	3.22	0.02	3.23	0.02	3.23	0.00	3.20	0.01	3.24	0.01	3.22	0.01	3.20	0.02
3	DDVPPRIARA	482	491	7	1109.606	2	3.31	0.03	3.59	0.01	3.51	0.02	4.45	0.05	4.79	0.02	4.72	0.03	4.55	0.01	4.81	0.01	4.81	0.02
4	DDVPPRIARAME	482	493	9	1369.689	2	3.95	0.03	3.99	0.02	3.93	0.04	5.92	0.10	6.00	0.04	5.95	0.06	6.19	0.02	6.21	0.03	6.19	0.02
5	PPRIARA	485	491	4	780.4839	2	2.47	0.04	2.51	0.01	2.44	0.01	3.51	0.04	3.56	0.02	3.52	0.02	3.64	0.01	3.62	0.03	3.65	0.03
6	ELEAATHNRPLIYL	504	517	12	1639.88	2	0.63	0.01	0.65	0.03	0.64	0.02	1.03	0.01	1.00	0.01	0.97	0.01	1.53	0.05	1.45	0.03	1.40	0.03
7	LEAATHNRPLIY	505	516	10	1397.754	2	0.55	0.02	0.52	0.02	0.48	0.02	0.84	0.01	0.77	0.01	0.75	0.01	1.27	0.02	1.22	0.02	1.18	0.02
8	LEAATHNRPLIYL	505	517	11	1510.838	2	0.49	0.01	0.49	0.01	0.51	0.02	0.83	0.01	0.84	0.01	0.81	0.02	1.28	0.03	1.28	0.02	1.20	0.03
9	ATHNRPLIY	508	516	7	1084.59	1	0.25	0.02	0.27	0.03	0.26	0.04	0.37	0.03	0.37	0.01	0.35	0.01	0.57	0.02	0.55	0.02	0.48	0.03
10	ATHNRPLIYL	508	517	8	1197.674	2	0.18	0.01	0.21	0.02	0.19	0.02	0.34	0.01	0.34	0.01	0.35	0.03	0.49	0.01	0.50	0.02	0.53	0.01
11	QIIEANY	543	549	6	850.4305	1	0.39	0.02	0.40	0.04	0.37	0.03	0.79	0.02	0.82	0.02	0.77	0.03	1.33	0.04	1.32	0.02	1.29	0.03
12	ANYHSSNPYHNSTHSADVL	547	565	17	2113.932	3	1.53	0.05	1.49	0.03	1.50	0.01	1.86	0.02	1.81	0.02	1.77	0.05	2.16	0.01	2.15	0.02	2.12	0.04
13	NYHSSNPYHNSTHSADVL	548	565	16	2042.895	3	1.31	0.05	1.36	0.02	1.37	0.02	1.66	0.01	1.65	0.01	1.62	0.03	1.95	0.02	1.90	0.01	1.87	0.01
14	HSSNPYHNSTHSADVL	550	565	14	1765.789	2	1.01	0.07	1.06	0.06	1.06	0.04	1.27	0.01	1.32	0.02	1.30	0.01	1.54	0.01	1.58	0.01	1.53	0.01
15	SADVLHATAYFLSKER	561	576	15	1807.934	2	2.49	0.09	2.64	0.06	2.68	0.05	3.07	0.04	3.27	0.12	3.10	0.05	3.50	0.04	3.56	0.04	3.52	0.04
16	FLSKERIKE	571	579	8	1149.663	2	1.43	0.06	1.43	0.03	1.48	0.01	1.61	0.04	1.78	0.02	1.70	0.05	1.89	0.01	1.99	0.02	1.98	0.02
17	FLSKERIKETLDPIDE	571	586	14	1933.028	3	2.92	0.06	2.87	0.06	2.86	0.03	3.48	0.02	3.47	0.01	3.42	0.02	4.12	0.03	4.03	0.01	3.99	0.02
18	LSKERIKETLDPIDE	572	586	13	1785.959	2	2.63	0.06	2.70	0.04	2.68	0.02	3.16	0.01	3.30	0.02	3.21	0.03	3.65	0.02	3.75	0.02	3.71	0.03
19	RIKETLDPIDE	576	586	9	1328.706	2	1.29	0.08	1.37	0.08	1.33	0.06	1.70	0.03	1.82	0.04	1.79	0.03	2.07	0.02	2.15	0.02	2.11	0.02
20	LIAATIHDVDHPGRTNSF	590	607	16	1963.998	3	2.66	0.02	2.59	0.01	2.57	0.03	3.60	0.04	3.61	0.01	3.54	0.01	4.17	0.02	4.12	0.02	4.06	0.05
21	AATIHDVDHPGRTNSF	592	607	14	1737.83	3	2.53	0.04	2.50	0.00	2.49	0.01	3.22	0.03	3.26	0.01	3.20	0.02	3.69	0.02	3.66	0.01	3.63	0.03
22	ATIHDVDHPGRTNSF	593	607	13	1666.793	2	2.60	0.05	2.58	0.04	2.58	0.02	3.30	0.04	3.42	0.02	3.36	0.02	3.73	0.02	3.84	0.02	3.78	0.03
23	LCNAGSE	608	614	6	693.2872	1	2.20	0.01	2.24	0.02	2.16	0.01	2.64	0.03	2.66	0.02	2.64	0.02	2.76	0.02	2.72	0.03	2.68	0.03
24	ESHHAAL	626	632	6	764.3686	1	0.36	0.02	0.37	0.03	0.37	0.03	0.82	0.01	0.94	0.01	0.87	0.01	1.08	0.01	1.21	0.01	1.17	0.01
25	AFQLTTGDD	633	641	8	967.4367	1	1.86	0.01	1.85	0.02	1.75	0.01	2.91	0.02	3.01	0.03	2.94	0.01	3.30	0.01	3.38	0.00	3.36	0.02
26	FQLTTGDD	634	641	7	896.3996	1	1.74	0.02	1.69	0.02	1.62	0.01	2.55	0.03	2.55	0.01	2.51	0.01	2.80	0.02	2.76	0.01	2.74	0.02
27	FQLTTGDDKCNIF	634	646	12	1501.699	2	2.60	0.02	2.72	0.01	2.68	0.01	4.13	0.05	4.20	0.03	4.10	0.01	4.72	0.03	4.79	0.01	4.74	0.05
28	KNMERNDYRTLRQ	647	659	12	1723.866	2	3.28	0.07	3.56	0.02	3.51	0.04	4.35	0.08	4.71	0.03	4.58	0.05	5.09	0.02	5.38	0.03	5.38	0.06
29	MTKHFEHVNKF	670	680	10	1417.705	2	1.35	0.04	1.35	0.04	1.35	0.06	2.15	0.07	2.29	0.04	2.19	0.04	2.36	0.04	2.49	0.04	2.49	0.04
30	MTKHFEHVNKFVNS	670	683	13	1717.848	3	1.87	0.08	1.70	0.01	1.64	0.02	3.27	0.02	3.52	0.01	3.37	0.02	3.86	0.02	3.94	0.01	3.92	0.05
31	MTKHFEHVNKFVNSINKPLAPLEE	670	693	21	2822.466	3	6.99	0.11	7.12	0.04	6.92	0.06	9.25	0.11	9.53	0.03	9.34	0.03	9.98	0.09	10.10	0.03	10.02	0.04
32	INKPLAPLEE	684	693	7	1123.636	2	4.01	0.01	3.90	0.02	3.88	0.02	4.00	0.02	3.94	0.02	3.92	0.01	4.06	0.01	3.94	0.03	3.95	0.01
33	INKPLAPLEENGETDKNQEVINT	684	706	20	2566.3	3	11.41	0.04	11.21	0.06	11.26	0.09	11.38	0.05	11.27	0.02	11.22	0.01	11.37	0.01	11.23	0.02	11.19	0.03
34	NGETDKNQEVINT	694	706	12	1461.682	2	6.56	0.04	6.50	0.05	6.56	0.07	6.47	0.03	6.55	0.02	6.47	0.03	6.50	0.01	6.50	0.03	6.48	0.03
35	LRTPENRT	708	715	6	986.5378	2	2.49	0.03	2.60	0.04	2.54	0.03	3.05	0.05	3.21	0.02	3.14	0.03	3.10	0.07	3.27	0.02	3.28	0.06
36	LRTPENRTLIKRML	708	721	12	1741.027	4	2.32	0.06	2.37	0.08	2.23	0.03	3.04	0.13	2.97	0.02	2.96	0.06	3.39	0.02	3.22	0.01	3.16	0.13
37	RTPENRTLI	709	717	7	1099.622	2	2.74	0.01	3.02	0.01	2.97	0.02	3.48	0.04	3.73	0.02	3.70	0.02	3.68	0.01	3.83	0.01	3.80	0.02
38	ADVSNPCRPLQ	725	735	8	1199.584	2	2.15	0.05	2.11	0.06	2.06	0.04	2.48	0.02	2.45	0.01	2.42	0.01	2.57	0.00	2.51	0.01	2.51	0.02
39	ADVSNPCRPLQY	725	736	9	1362.647	2	2.25	0.08	2.20	0.06	2.14	0.05	2.66	0.02	2.67	0.01	2.60	0.01	2.99	0.02	2.92	0.02	2.85	0.03
40	EWAARISE	739	746	7	961.4738	2	0.35	0.01	0.42	0.01	0.38	0.01	0.79	0.02	0.87	0.02	0.81	0.02	1.28	0.02	1.33	0.03	1.25	0.02
41	YFSQTDE	748	754	6	889.3574	1	1.53	0.01	1.52	0.02	1.42	0.02	2.53	0.04	2.54	0.01	2.50	0.03	2.66	0.00	2.63	0.01	2.63	0.01
42	YFSQTDEEKQQGLPVVM	748	764	15	1998.948	2	6.10	0.02	6.03	0.04	5.82	0.07	7.81	0.14	7.87	0.08	7.81	0.05	8.06	0.03	7.98	0.03	7.95	0.05
43	FSQTDEEKQQGLPVVM	749	764	14	1835.884	2	6.09	0.02	6.05	0.01	5.88	0.04	7.74	0.13	7.78	0.04	7.70	0.04	8.06	0.04	8.05	0.03	8.00	0.05
44	DEEKQQGLPVVM	753	764	10	1372.678	1	5.02	0.02	4.99	0.04	4.87	0.01	6.06	0.06	6.04	0.03	5.98	0.03	6.14	0.03	6.09	0.03	6.07	0.04
45	PVFDRNTCSIPKSQISF	765	781	14	1938.974	3	6.46	0.10	6.01	0.05	5.59	0.09	7.50	0.14	7.41	0.12	7.23	0.11	7.68	0.06	7.53	0.05	7.48	0.10
46	SFIDYFITDM	780	789	9	1251.56	1	3.24	0.07	3.16	0.04	3.03	0.06	3.79	0.05	3.86	0.03	3.83	0.03	3.81	0.03	3.85	0.05	3.90	0.07
47	FVDLPDL	796	802	5	818.4294	1	0.93	0.01	0.93	0.01	0.91	0.01	1.29	0.01	1.26	0.01	1.25	0.01	1.46	0.01	1.42	0.01	1.39	0.01
48	MQHLDNNF	803	810	7	1018.441	1	0.57	0.04	0.50	0.03	0.63	0.05	0.96	0.01	0.98	0.02	0.93	0.02	1.22	0.01	1.25	0.03	1.14	0.01
49	FKYWKGLDE	810	818	8	1185.594	1	1.85	0.03	1.93	0.02	1.73	0.02	2.56	0.05	2.63	0.04	2.53	0.04	2.94	0.04	3.11	0.02	3.01	0.04

Supplementary table 2: Deuterium exchange uptake for peptides from PDE8 T690P during interactions with RIα WT. Uptake is in Deuterons and SD is the standard deviation.

Sno.	Peptide	Start	End	Max Uptake (Deuterons)	MH+	Z	Number of deuterons exchanged at 1 min						Number of deuterons exchanged at 10 min						Number of deuterons exchanged at 30 min					
							RIα + PDE8 T690P		RIα + PDE8 T690P+		RIα		RIα + PDE8 T690P		RIα + PDE8 T690P+		RIα		RIα + PDE8 T690P		RIα + PDE8 T690P+ cAMP		RIα	
							Uptake	SD	Uptake	SD	Uptake	SD	Uptake	SD	Uptake	SD	Uptake	SD	Uptake	SD	Uptake	SD	Uptake	SD
1	LKDSIVQL	29	36	7	915.551	1	0.41	0.01	0.39	0.02	0.42	0.05	1.16	0.05	1.15	0.05	1.24	0.06	1.77	0.03	1.76	0.04	1.85	0.01
2	LEKEEAK	55	61	6	846.4567	2	1.47	0.04	1.34	0.04	0.92	0.03	2.33	0.03	2.24	0.03	1.34	0.05	2.72	0.02	2.71	0.03	1.63	0.02
3	EKEEAKQIQNL	56	66	10	1329.701	2	6.30	0.06	6.32	0.06	5.45	0.08	6.33	0.01	6.28	0.02	5.32	0.04	6.23	0.02	6.25	0.02	5.21	0.09
4	YVRKVIPKDYKTMAA	111	125	13	1782.994	3	6.41	0.06	6.26	0.05	5.99	0.17	7.58	0.04	7.40	0.04	6.91	0.11	7.71	0.01	7.62	0.04	7.10	0.10
5	LAKAIEKNVL	126	135	9	1098.688	1	3.32	0.01	3.26	0.01	3.27	0.08	4.45	0.04	4.33	0.03	4.13	0.10	5.41	0.06	5.33	0.06	4.92	0.05
6	AKAIEKNVL	127	135	8	985.6041	2	2.95	0.06	3.00	0.03	2.78	0.05	3.71	0.09	3.60	0.08	3.26	0.08	4.47	0.06	4.47	0.06	3.82	0.05
7	IAGETVIQQGDEGDN	157	171	14	1545.703	2	2.11	0.07	2.10	0.04	3.09	0.10	2.79	0.05	2.65	0.03	3.67	0.10	3.32	0.02	3.31	0.04	4.03	0.03
8	TVIQQGDEGDNF	161	172	11	1322.586	1	2.60	0.02	2.47	0.04	2.53	0.09	3.40	0.04	3.34	0.03	2.95	0.07	4.04	0.03	3.94	0.02	3.09	0.04
9	DVYVNNE	181	187	6	852.3734	1	1.27	0.02	1.26	0.01	0.93	0.03	1.91	0.05	2.03	0.02	1.29	0.04	2.77	0.06	2.65	0.03	1.49	0.01
10	WATSVGEGGSF	188	198	10	1097.49	1	3.08	0.02	2.95	0.02	2.93	0.08	4.06	0.03	3.89	0.01	3.25	0.06	4.89	0.03	4.81	0.05	3.39	0.03
11	IYGTPRAATVKAKTNVKL	204	221	16	1931.144	3	2.07	0.04	1.76	0.05	1.70	0.07	4.32	0.05	3.89	0.03	2.48	0.14	6.89	0.07	6.52	0.04	3.46	0.10
12	VKAKTNVKL	213	221	8	1000.651	2	0.61	0.03	0.59	0.02	0.77	0.03	1.26	0.01	1.14	0.01	1.07	0.05	2.20	0.03	2.13	0.05	1.46	0.03
13	WGIDRDSY	222	229	7	1011.453	1	0.90	0.03	0.85	0.02	0.55	0.03	1.54	0.00	1.42	0.02	0.86	0.03	1.98	0.01	1.91	0.02	1.03	0.01
14	RRILMGST	230	237	7	933.5298	2	2.10	0.01	2.04	0.01	1.70	0.05	2.72	0.01	2.61	0.03	2.21	0.05	3.26	0.02	3.20	0.03	2.75	0.02
15	RRILMGSTL	230	238	8	1046.614	2	2.20	0.03	2.08	0.04	1.76	0.07	3.23	0.01	3.14	0.02	2.62	0.09	3.84	0.03	3.80	0.02	3.20	0.03
16	RKRKMYEEF	239	247	8	1286.667	2	2.06	0.02	1.85	0.03	1.70	0.08	3.28	0.03	3.22	0.03	2.67	0.11	3.49	0.02	3.46	0.03	3.05	0.04
17	FLSKVSIL	247	254	7	906.5659	2	2.99	0.03	2.82	0.02	2.16	0.06	3.96	0.04	3.92	0.03	2.95	0.09	4.39	0.06	4.33	0.03	3.35	0.05
18	LSKVSIL	248	254	6	759.4975	1	2.40	0.01	2.31	0.02	1.81	0.05	3.42	0.03	3.34	0.03	2.62	0.08	3.72	0.03	3.71	0.02	3.01	0.04
19	ESLDKWERL	255	263	8	1175.606	2	1.92	0.01	1.81	0.01	1.55	0.06	3.12	0.06	3.05	0.04	2.20	0.07	3.49	0.01	3.49	0.02	2.59	0.02
20	TVADALE	264	270	6	718.3618	1	0.76	0.03	0.54	0.02	0.41	0.04	2.31	0.03	2.19	0.04	1.25	0.07	3.11	0.01	3.06	0.03	1.74	0.02
21	DALEPVQF	267	274	6	918.4567	1	1.32	0.03	1.21	0.03	0.83	0.04	2.18	0.08	1.99	0.03	1.40	0.05	2.53	0.05	2.58	0.05	1.65	0.02
22	PVQFEDGQKIVVQGEPGDEF	271	290	17	2218.066	2	3.64	0.07	3.55	0.07	3.02	0.10	5.29	0.01	5.02	0.01	3.86	0.06	6.83	0.03	6.63	0.08	4.56	0.02
23	IILEGSA	292	298	6	702.4032	1	0.83	0.01	0.84	0.01	0.87	0.02	1.13	0.01	1.11	0.01	1.06	0.03	1.45	0.01	1.42	0.01	1.41	0.00
24	QRRSENEEF	302	310	8	1194.55	2	3.48	0.06	3.50	0.06	2.99	0.04	3.45	0.01	3.42	0.01	2.94	0.03	3.42	0.01	3.40	0.02	2.92	0.07
25	FVEVGRLGPSDY	310	321	10	1338.669	2	1.76	0.03	1.73	0.02	1.31	0.04	2.52	0.03	2.45	0.02	1.83	0.05	3.24	0.03	3.12	0.02	2.02	0.02
26	VEVGRLGPSDY	311	321	9	1191.6	1	1.31	0.04	1.29	0.03	0.92	0.04	2.11	0.01	2.04	0.02	1.43	0.04	2.83	0.03	2.75	0.02	1.62	0.01
27	LMNRPRAAT	328	336	7	1029.562	2	1.19	0.02	1.03	0.01	0.92	0.04	2.30	0.04	2.14	0.02	1.42	0.08	3.36	0.05	3.18	0.03	1.74	0.06
28	MNRPRAAT	329	336	6	916.4781	2	0.88	0.04	0.76	0.03	0.53	0.04	2.04	0.03	1.86	0.02	1.07	0.08	3.04	0.03	2.98	0.04	1.36	0.07
29	VVARGPLKC	337	345	7	942.5553	1	0.54	0.05	0.53	0.04	0.44	0.04	1.18	0.01	1.13	0.01	1.02	0.04	1.92	0.02	1.86	0.01	1.62	0.01
30	VARGPLKC	338	345	6	843.4869	2	0.48	0.05	0.46	0.01	0.37	0.03	1.09	0.01	1.02	0.03	0.93	0.06	1.79	0.03	1.69	0.04	1.49	0.01
31	VKLDRPRF	346	353	6	1030.616	2	0.91	0.05	0.87	0.03	0.53	0.04	1.72	0.02	1.67	0.02	1.00	0.03	2.07	0.02	2.05	0.03	1.05	0.01
32	ERVLGPCSDIL	354	364	9	1201.625	2	2.66	0.07	2.45	0.04	2.29	0.10	3.55	0.04	3.40	0.03	2.79	0.08	4.24	0.06	4.15	0.05	3.07	0.08
33	KRNIQQYNSF	365	374	9	1297.665	2	4.57	0.06	4.52	0.07	4.24	0.16	5.16	0.02	5.10	0.04	4.69	0.08	5.18	0.02	5.20	0.03	4.80	0.09

Supplementary table 3. Deuterium exchange uptake for peptides from RIα WT during interactions with PDE8 T690P. Uptake is in Deuterons and SD is the standard deviation.

Sno.	Peptide	Start	End	Max Uptake (Deuterons)	MH+	Z	Number of deuterons exchanged at 1 min										Number of deuterons exchanged at 10 min										Number of deuterons exchanged at 30 min									
							RIα T207A		RIα T207A + cAMP		RIα T207A + PDE8		RIα T207A + PDE8 + cAMP		RIα T207A + PDE8 + 5'AMP		RIα T207A		RIα T207A + cAMP		RIα T207A + PDE8		RIα T207A + PDE8 + cAMP		RIα T207A + PDE8 + 5'AMP		RIα T207A		RIα T207A + cAMP		RIα T207A + PDE8		RIα T207A + PDE8 + cAMP		RIα T207A + PDE8 + 5'AMP	
							Uptake	SD	Uptake	SD	Uptake	SD	Uptake	SD	Uptake	SD	Uptake	SD	Uptake	SD	Uptake	SD	Uptake	SD	Uptake	SD	Uptake	SD	Uptake	SD	Uptake	SD	Uptake	SD	Uptake	SD
1	DEISPPPPNPVVKGRRRRGAIS	78	99	16	2398.3426	4	8.13	0.08	8.18	0.13	8.59	0.19	8.54	0.25	8.13	0.21	7.99	0.08	7.97	0.03	8.43	0.11	8.37	0.07	7.88	0.08	8.04	0.06	7.94	0.02	8.32	0.09	8.70	0.24	7.91	0.01
2	DEISPPPPNPVVKGRRRRGAISA	78	100	17	2469.3797	4	8.36	0.10	8.46	0.09	8.87	0.20	8.81	0.17	8.46	0.16	8.28	0.07	8.29	0.05	8.67	0.10	8.71	0.02	8.12	0.05	8.30	0.05	8.36	0.02	8.42	0.03	8.90	0.01	8.21	0.08
3	DEISPPPPNPVVKGRRRRGAISAE	78	101	18	2598.4223	4	9.37	0.10	9.36	0.17	9.98	0.24	9.80	0.26	9.41	0.24	9.21	0.08	9.19	0.06	9.65	0.10	9.58	0.08	9.09	0.06	9.21	0.05	9.24	0.02	9.49	0.05	9.88	0.21	9.14	0.04
4	YVRKVIPKDYKTMAA	111	125	13	1782.9935	4	5.82	0.09	5.69	0.06	5.61	0.07	5.09	0.07	5.02	0.11	7.04	0.06	6.96	0.10	7.06	0.06	6.55	0.15	6.11	0.12	7.33	0.02	7.37	0.02	7.41	0.04	7.14	0.01	6.87	0.05
5	YVRKVIPKDYKTMAAL	111	126	14	1896.0776	4	6.23	0.07	6.05	0.03	6.14	0.06	5.56	0.04	5.60	0.05	7.57	0.05	7.36	0.04	7.56	0.08	6.91	0.11	6.66	0.11	7.99	0.04	7.81	0.03	8.20	0.06	7.51	0.01	7.50	0.09
6	LAKAIEKNVL	126	135	9	1098.6881	3	2.94	0.04	2.94	0.03	3.08	0.02	2.98	0.03	2.95	0.04	3.71	0.02	3.66	0.05	3.66	0.01	3.35	0.05	3.18	0.07	4.47	0.03	4.48	0.01	4.47	0.02	3.79	0.00	3.65	0.02
7	FSHLDDNE	136	143	7	976.4007	2	1.57	0.03	1.51	0.04	1.69	0.04	1.60	0.03	1.39	0.07	1.58	0.02	1.53	0.04	1.67	0.05	1.60	0.04	1.46	0.03	1.54	0.02	1.55	0.02	1.65	0.02	1.56	0.07	1.59	0.20
8	FSHLDDNERSDIF	136	148	12	1594.7132	3	2.10	0.02	2.10	0.03	2.22	0.02	2.14	0.02	1.78	0.04	2.26	0.03	2.21	0.02	2.45	0.05	2.32	0.07	2.07	0.03	2.34	0.01	2.28	0.02	2.59	0.02	2.33	0.13	2.17	0.04
9	IAGETVIQQGDEGDNF	157	172	15	1692.7711	2	3.08	0.07	2.88	0.02	3.22	0.01	2.88	0.02	2.60	0.04	3.68	0.02	3.52	0.07	3.98	0.04	3.49	0.07	3.25	0.07	3.96	0.01	3.86	0.02	4.68	0.02	3.51	0.13	3.55	0.02
10	YVIDQGEM	173	180	7	954.4237	1	0.82	0.02	0.76	0.02	0.82	0.06	0.77	0.03	0.62	0.03	0.93	0.02	0.89	0.02	1.05	0.05	0.90	0.02	0.92	0.02	1.03	0.01	0.96	0.02	1.34	0.02	0.95	0.03	0.99	0.03
11	YVIDQGEMDV	173	182	9	1168.5191	2	0.73	0.02	0.71	0.02	0.75	0.05	0.72	0.04	0.57	0.02	0.90	0.01	0.80	0.01	1.06	0.04	0.84	0.01	0.85	0.02	1.02	0.01	0.89	0.01	1.68	0.02	0.82	0.03	0.96	0.03
12	DVYVNNE	181	187	6	852.3734	1	1.14	0.03	1.10	0.01	1.21	0.03	1.16	0.02	1.10	0.01	1.50	0.01	1.40	0.02	1.54	0.02	1.34	0.03	1.22	0.01	1.70	0.02	1.61	0.02	2.02	0.02	1.66	0.18	1.45	0.04
13	WATSVGEGGSF	188	198	10	1097.4898	1	2.66	0.06	2.52	0.02	2.80	0.02	2.57	0.02	2.22	0.05	3.03	0.03	2.82	0.04	3.40	0.02	2.92	0.04	2.69	0.05	3.18	0.01	2.93	0.02	4.05	0.06	3.00	0.10	2.94	0.02
14	FGELALI	198	204	6	762.4396	1	0.12	0.03	0.12	0.03	0.23	0.07	0.14	0.03	0.16	0.03	0.20	0.03	0.15	0.03	0.31	0.03	0.15	0.03	0.21	0.04	0.43	0.05	0.14	0.03	N/A	N/A	0.23	0.10	0.24	0.03
15	ALIYGAPRAATVKAKTNVKL	202	221	18	2085.2543	4	2.24	0.08	1.97	0.06	2.28	0.06	2.03	0.03	1.91	0.03	3.23	0.09	2.77	0.05	3.73	0.06	2.68	0.04	2.69	0.09	4.06	0.07	3.28	0.03	5.66	0.13	2.94	0.14	3.29	0.06
16	YGAPRAATVKAKTNVKL	205	221	15	1788.049	3	1.20	0.07	1.13	0.05	1.33	0.03	1.28	0.11	1.17	0.06	1.80	0.10	1.57	0.04	2.40	0.02	1.61	0.01	1.66	0.09	2.36	0.05	1.99	0.04	3.51	0.15	1.86	0.08	2.11	0.04
17	GAPRAATVKAKTNVKL	206	221	14	1624.9857	3	1.19	0.06	1.20	0.08	1.40	0.06	1.35	0.06	1.18	0.06	1.94	0.07	1.62	0.04	2.40	0.10	1.70	0.04	1.68	0.07	2.56	0.09	1.98	0.03	3.73	0.15	1.90	0.04	2.04	0.06
18	VKAKTNVKL	213	221	8	1000.6513	2	0.67	0.07	0.63	0.03	0.67	0.03	0.61	0.02	0.70	0.05	0.91	0.02	0.85	0.03	0.97	0.02	0.74	0.03	0.81	0.03	1.17	0.02	1.12	0.02	1.47	0.05	0.92	0.01	0.92	0.03
19	WGIDRDSY	222	229	7	1011.453	2	0.67	0.03	0.66	0.05	0.76	0.05	0.72	0.04	0.65	0.03	1.00	0.00	1.02	0.03	1.14	0.02	1.01	0.02	0.87	0.01	1.22	0.01	1.26	0.01	1.52	0.01	1.20	0.00	1.07	0.04
20	WGIDRDSYRRILMGSTL	222	238	16	2039.0491	4	2.16	0.08	2.05	0.06	2.15	0.05	2.04	0.03	1.76	0.03	3.36	0.08	3.12	0.04	3.33	0.02	3.02	0.08	2.41	0.05	4.33	0.05	4.00	0.03	4.74	0.02	3.86	0.01	3.01	0.05
21	RRILMGSTL	230	238	8	1046.6139	2	1.87	0.04	1.83	0.01	1.79	0.03	1.72	0.02	1.56	0.02	2.69	0.04	2.53	0.05	2.58	0.02	2.40	0.05	2.02	0.05	3.28	0.02	3.17	0.04	3.15	0.02	2.89	0.01	2.48	0.03
22	FLSKVSIL	247	254	7	906.5659	2	2.71	0.03	2.60	0.01	2.62	0.02	2.50	0.01	2.20	0.04	3.49	0.02	3.32	0.07	3.37	0.02	3.23	0.04	2.80	0.07	3.94	0.01	3.86	0.02	3.86	0.01	3.61	0.21	3.28	0.01
23	LSKVSIL	248	254	6	759.4975	2	2.06	0.03	2.04	0.03	2.15	0.01	2.09	0.01	1.90	0.03	2.80	0.02	2.75	0.08	2.83	0.01	2.69	0.04	2.26	0.05	3.23	0.02	3.29	0.02	3.32	0.01	3.14	0.15	2.74	0.02
24	ESLDKWERLTVA	255	266	11	1446.7587	2	1.46	0.05	1.43	0.05	1.47	0.02	1.43	0.02	1.37	0.01	1.99	0.03	1.95	0.05	1.94	0.03	1.81	0.03	1.60	0.04	2.39	0.03	2.48	0.01	2.13	0.05	2.09	0.01	1.91	0.02
25	ESLDKWERLTVAD	255	267	12	1561.7857	3	1.81	0.02	1.78	0.01	1.51	0.03	1.47	0.03	1.64	0.05	2.50	0.03	2.36	0.05	2.10	0.01	1.79	0.05	2.02	0.02	3.02	0.04	2.89	0.03	2.41	0.06	2.37	0.14	2.49	0.09
26	ESLDKWERLTVADALE	255	270	15	1874.9494	3	1.91	0.04	1.85	0.02	1.81	0.03	1.76	0.02	1.68	0.01	3.11	0.06	2.82	0.07	3.23	0.06	2.71	0.05	2.19	0.04	4.03	0.05	3.87	0.05	3.93	0.01	3.32	0.09	2.93	0.03
27	LDKWERL	257	263	6	959.5309	2	1.19	0.05	1.16	0.03	1.19	0.06	1.08	0.07	1.12	0.02	1.64	0.01	1.57	0.05	1.59	0.03	1.43	0.05	1.30	0.02	1.89	0.01	1.87	0.01	2.01	0.06	1.62	0.13	1.56	0.03
28	TVADALE	264	270	6	718.3618	1	0.41	0.01	0.39	0.02	0.54	0.05	0.42	0.05	0.34	0.03	1.12	0.02	1.01	0.02	1.21	0.04	0.87	0.04	0.67	0.02	1.47	0.01	1.37	0.01	2.07	0.04	1.24	0.02	1.06	0.04
29	FEDGQKIVVQGEPGDEF	274	290	15	1893.8865	2	2.01	0.03	1.85	0.03	1.92	0.05	1.87	0.04	1.91	0.02	2.48	0.08	2.29	0.07	2.32	0.05	2.14	0.04	2.09	0.03	3.07	0.03	2.76	0.05	3.34	0.04	2.53	0.18	2.34	0.03
30	EDGQKIVVQGEPGDEF	275	290	14	1746.8181	2	1.97	0.04	1.89	0.02	2.12	0.03	1.99	0.04	1.94	0.02	2.41	0.04	2.26	0.02	2.56	0.03	2.22	0.06	2.07	0.01	2.95	0.03	2.72	0.01	3.44	0.03	2.55	0.10	2.40	0.04
31	IILEGSA	292	298	6	702.4032	1	0.83	0.03	0.81	0.01	0.83	0.02	0.78	0.01	0.72	0.02	1.01	0.02	0.95	0.02	0.94	0.02	0.87	0.02	0.88	0.02	1.34	0.03	1.20	0.02	1.15	0.01	0.97	0.11	0.99	0.02
32	QRRSENEE	302	309	7	1047.4814	2	2.64	0.06	2.73	0.04	2.92	0.04	2.79	0.06	2.55	0.07	2.64	0.02	2.62	0.07	2.93	0.03	2.74	0.06	2.54	0.02	2.72	0.01	2.54	0.02	2.91	0.04	2.70	0.07	2.55	0.04
33	FVEVGRLGPSDY	310	321	10	1338.6688	2	1.66	0.05	1.58	0.03	1.65	0.04	1.58	0.03	1.53	0.02	2.20	0.01	2.13	0.03	2.28	0.02	2.10	0.03	1.90	0.02	2.37	0.01	2.33	0.02	2.65	0.02	2.34	0.06	2.23	0.03
34	VEVGRLGPSDYFGE	311	324	12	1524.7329	2	0.94	0.03	0.84	0.03	0.99	0.06	0.94	0.05	0.84	0.02	1.41	0.01	1.32	0.02	1.58	0.01	1.37	0.02	1.15	0.02	1.63	0.02	1.47	0.01	1.87	0.02	1.46	0.04	1.46	0.03
35	MNRPRAAT	329	336	6	916.4781	2	0.85	0.03	0.85	0.03	0.99	0.05	0.90	0.03	0.86	0.03	1.21	0.03	1.15	0.03	1.49	0.04	1.13	0.03	1.02	0.03	1.52	0.05	1.32	0.03	2.14	0.03	1.34	0.03	1.28	0.05
36	NRPRAAT	330	336	5	785.4377	2	0.88	0.07	0.86	0.06	0.96	0.05	0.92	0.06	0.86	0.03	1.31	0.01	1.18	0.01	1.51	0.02	1.18	0.03	1.04	0.01	1.56	0.04	1.37	0.01	2.13	0.02	1.34	0.01	1.28	0.02
37	VARGPLKC	338	345	6	843.4869	2	0.46	0.05	0.53	0.05	0.52	0.09	0.58	0.07	0.49	0.05	0.97	0.02	0.94	0.02	0.84	0.02	0.81	0.02	0.70	0.02	1.50	0.03	1.43	0.03	1.29	0.04	1.17	0.02	0.88	0.03
38	ARGPLKC	339	345	5	744.4185	2	0.35	0.04	0.37	0.05	0.40	0.07	0.42	0.05	0.35	0.04	0.79	0.01	0.78	0.02	0.72	0.03	0.69	0.02	0.56	0.01	1.27	0.02	1.23	0.01	1.19	0.02	1.01	0.02	0.83	0.03
39	VKLDRPRF	346	353	6	1030.6156	2	0.60	0.04	0.60	0.03	0.74	0.04	0.63	0.03	0.53	0.03	1.07	0.03	1.10	0.05	1.21	0.06	1.08	0.03	0.83	0.03	1.19	0.03	1.31	0.04	1.47	0.07	1.15	0.03	1.13	0.06
40	KLDRPRF	347	353	5	931.5472	2	0.62	0.02	0.62	0.03	0.72	0.02	0.67	0.02	0.57	0.02	1.10	0.02	1.17	0.04	1.19	0.05	1.14	0.03	0.86	0.03	1.16	0.01	1.31	0.03	1.45	0.02	1.26	0.03	1.18	0.05
41	ERVLGPCSD	354	362	7	975.4564	2	1.89	0.02	1.80	0.01	1.96	0.03	1.84	0.01	1.49	0.03	2.32	0.01	2.27	0.04	2.43	0.05	2.29	0.04	2.02	0.03	2.44	0.02	2.46	0.01	2.78	0.05	2.39	0.08	2.27	0.04
42	ERVLGPCSDIL	354	364	9	1201.6245	2	2.40	0.03	2.32	0.04	2.43	0.04	2.26	0.04	1.98	0.05	2.88	0.03	2.80	0.03	2.92	0.04	2.74	0.05	2.44	0.04	3.16	0.04	3.02	0.03	3.62	0.04	2.94	0.13	2.74	0.04
43	ILKRNIQQ	363	370	7	1012.6262	2	2.29	0.06	2.24	0.03	2.20	0.02	2.12	0.06	1.78	0.08	3.36	0.06	3.22	0.07	3.23	0.02	3.05	0.05	2.58	0.06	3.82	0.02	3.77	0.04	3.78	0.03	3.69	0.02	3.17	0.05
44	ILKRNIQQY	363	371	8	1175.6895	2	2.52	0.04	2.46	0.03	2.44	0.06	2.31	0.03	1.79	0.05	3.73	0.03	3.57	0.06	3.59	0.03	3.45	0.04	2.84	0.11	4.19	0.05	4.14	0.03	4.11	0.05	3.99	0.03	3.50	0.03
45	ILKRNIQQYNSF	363	374	11	1523.8329	2	4.58	0.02	4.52	0.06	4.42	0.04	4.26	0.05	3.73	0.08	6.05	0.02	5.80	0.07	5.78	0.04	5.58	0.08	4.90	0.11	6.51	0.04	6.37	0.04	6.30	0.01	6.18	0.01	5.74	0.07
46	KRNIQQYNSF	365	374	9	1297.6648	2	3.93	0.04	3.91	0.03	3.94	0.03	3.84	0.03	3.41	0.06	4.53	0.04	4.47	0.03	4.52	0.03	4.48	0.05	4.18	0.08	4.77	0.04	4.71	0.03	4.66	0.03	4.80	0.00	4.56	0.04

Supplementary table 4. Deuterium exchange uptake for peptides from RIα T207A during interactions with PDE8 WT. Uptake is in Deuterons and SD is the standard deviation.
